# Aqueous extract of *Sargentodoxa cuneata* alleviates ulcerative colitis and its associated liver injuries in mice through the modulation of intestinal flora and related metabolites

**DOI:** 10.3389/fmicb.2024.1295822

**Published:** 2024-01-24

**Authors:** Feng Xu, Piao Yu, Hongmei Wu, Mei Liu, Hongyun Liu, Qian Zeng, Dengli Wu, Xiangpei Wang

**Affiliations:** ^1^Department of Pharmacy, Guizhou University of Traditional Chinese Medicine, Guiyang, China; ^2^School of Chinese Ethnic Medicine, Guizhou Minzu University, Guiyang, China

**Keywords:** *Sargentodoxa cuneata*, ulcerative colitis, ulcerative colitis-associated liver injury, intestinal flora, short-chain fatty acid, organic acid

## Abstract

**Background:**

Ulcerative colitis (UC) is a refractory disease worldwide. Liver injury can be found clinically with UC, and now, it is found that gut dysbiosis is an important mechanism in the pathogenesis of UC. *Sargentodoxa cuneata* has been used as a traditional Chinese medicine and is commonly used clinically for the treatment of UC. The main objective of this study was to investigate the intrinsic mechanisms of *Sargentodoxa cuneata* in the treatment of UC and its associated liver injuries from the perspective of intestinal flora and related metabolites.

**Methods:**

Ultra-performance liquid chromatography-mass spectrometry was used to identify the components in the aqueous extract of *Sargentodoxa cuneata* (AESc). Mice with UC induced by dextran sulfate sodium were used to study the effects of AESc on UC and its associated liver injuries. Furthermore, 16S rRNA gene sequencing and analysis were performed on intestinal contents, and correlation analysis of intestinal flora with short-chain fatty acids (SCFAs) and organic acids was performed.

**Results:**

A total of 114 compounds were identified in AESc. AESc improved disease activity index scores, liver index, and colon length in mice with UC and had a good protective effect on intestine and liver injuries. Moreover, the administration of AESc regulated gut microbiota dysbiosis and the levels of a few SCFAs and organic acids in mice with UC. In addition, the correlation analysis results showed that the *Megamonas* and *Bifidobacterium* were the key intestinal flora related to the levels of differential SCFAs and organic acids in mice with UC after AESc intervention.

**Conclusion:**

AESc has a good protective effect on UC and UC related liver injuries. Modulation of the intestinal flora and its metabolites (SCFAs and a few organic acids) is an important pathway for AESc in the treatment of UC and also provides a rationale for the clinical use of *Sargentodoxa cuneata* in the treatment of UC.

## Graphical abstract

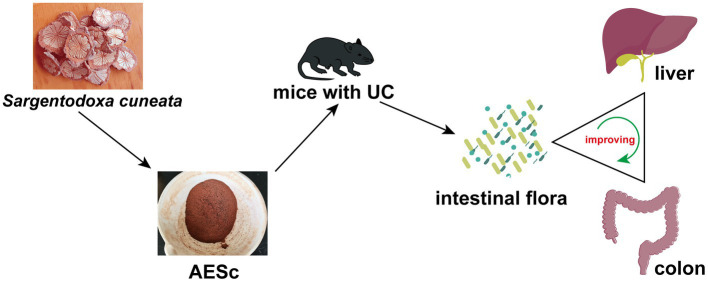

## 1 Introduction

Ulcerative colitis (UC) is a chronic intestinal immunoinflammatory disease, which is classified as one of the refractory diseases by the World Health Organization. UC clinical manifestations include abdominal pain, diarrhea, mucopurulent, and bloody stool (Le Berre et al., 2023). With changes in lifestyle and dietary habits, the prevalence of UC is rapidly increasing worldwide (Lopes et al., 2023). At present, UC therapeutic drugs are ineffective in relieving the disease, expensive, easy to relapse after stopping the drug, and have many adverse reactions. Therefore, it is still urgent to find effective, safe, and inexpensive UC therapeutic drugs. Traditional medicine is a treasure trove for the discovery of medicines for UC and has identified a variety of potential ingredients for the treatment of UC (Wang M. et al., 2023). Miao medicine has the characteristics of good efficacy, simultaneous treatment of manifestation and root cause of disease, small toxic side effects, and low price, which has attracted much attention (Jiang et al., 2019).

*Sargentodoxa cuneata* is the dried vine stem of the plant Da Xue Teng [*Sargentodoxa cuneata* (Oliv.) Rehd. et Wils.], which has the traditional function of clearing heat and removing toxins, invigorating blood circulation, dispelling wind, and relieving pain, and it can clear heat-, wind-, and dampness-poison to improve the clinical symptoms of UC, such as belly pain, diarrhea with pus, and blood and abdominal distension (Commission SP, 2020). Granules of *Sargentodoxa cuneata* has been reported to have anti-UC effects, and our group pre-discovered that aqueous extract of *Sargentodoxa cuneata* (AESc) and ethyl acetate extract of AESc have a good therapeutic effect on UC (Wang Y. et al., 2023; Yu et al., 2023), providing new options for the treatment of UC, but pharmacological mechanism of *Sargentodoxa cuneata* in the treatment of UC is not fully elaborated.

The intestinal flora is mainly composed of probiotics, neutral bacteria, and pathogenic bacteria, and the most abundant intestinal flora is colonized in the colon (Metwaly et al., 2022). In normal conditions, the intestinal flora maintains a dynamic balance in the organism, whereas there are significant differences in both species abundance and biodiversity of the intestinal flora in patients with UC, with an overall decrease in probiotics and an increase in pathogenic bacteria (Matsuoka, 2021). Modern studies show that the intestinal flora and its metabolites are closely related to the development of UC and its associated liver injuries (Liu et al., 2020). Studies have confirmed that regulating intestinal flora is an important way to treat UC using traditional Chinese medicine (Liu et al., 2022).

In this study, we first determined the effects of AESc on UC and its associated liver injuries using a dextran sulfate sodium (DSS)-induced UC mouse model. On this basis, the effects of AESc on the intestinal flora and its metabolites [short-chain fatty acids (SCFAs) and a few organic acids] were evaluated. Furthermore, the correlation of gut flora with differential SCFAs and organic acids was studied. It is expected that our study will provide new ideas for elucidating the pharmacological mechanism of AESc in the treatment of UC and its related liver injuries, as well as for the clinical application of AESc.

## 2 Materials and methods

### 2.1 Chemicals and reagents

DSS (molecular weight 36–50 kDa) was purchased from MP Biomedical (Illkirch, France). Formaldehyde was obtained from Chongqing Jiangchuan Chemical Group Co. Ltd. (Chongqing, China). Acetonitrile and methanol were purchased from Thermo Fisher Scientific (FairLawn, NJ, United States), and formic acid was provided by Sigma–Aldrich (St. Louis, MO, United States). HE dye kit was purchased from Leagene Inc. (Beijing, China). The short-chain fatty acid and organic acid standards were purchased from Sigma–Aldrich (St. Louis, MO, United States), and the purity was ≥99.9%.

### 2.2 Preparation and composition of AESc

*Sargentodoxa cuneata* was purchased from Hubei Province in China, identified by Prof. Wang Xiangpei of Guizhou Minzu University as the dried vine stem of *Sargentodoxa cuneata* (Oliv.) Rehd. et Wils. The quality of the medicinal materials of *Sargentodoxa cuneata* met the requirements of the Chinese Pharmacopoeia (2020) (Commission SP, 2020). The dried herb of *Sargentodoxa cuneata* was weighed and soaked in water for 15 min, and the water submerged the herb up to 2–3 cm. We heated the decoction over high heat until boiling, then reduced the heat and cooked for 15 min, and filtered the obtained decoction. The dregs were added with water and then decocted twice according to the above process. Finally, the filtered solutions were combined and concentrated using a rotary evaporator to obtain the AESc extract and stored in a − 4°C refrigerator until use. The stability of the AESc extract was controlled using the high-performance liquid chromatography (HPLC) fingerprint method (Supplementary Figure S1).

The composition of AESc was analyzed by ultra-performance liquid chromatography-mass spectrometry (UPLC–MS). For UPLC separation, a C_18_ analytical column (150 × 2.1 mm, 1.8 μm) was used. The mobile phase was acetonitrile (solvent B) and 0.1% (v/v) formic acid aqueous solution (solvent A). The gradient elution program was as follows: 0–10 min, 5–70% B, 10–17 min; 70–100% B, 17–18 min; 100–100% B, 18–19 min; 100–5% B, 19–21 min; and 5–5% B. The flow rate was 0.3 mL/min. The column oven was set at 40°C, and the injection volume was 1 μL.

Electrospray ionization (ESI) was used in positive and negative ion modes, respectively. The source temperature was set at 500°C (positive ions) and 450°C (negative ions), ionspray voltage floating (ISVF) was set at 5500 V (positive ions) and 4,400 V (negative ions), and time-of-flight mass spectrometry (TOF-MS) scan range was set at 100–1200 Da. Secondary mass spectra were obtained by information dependent acquisition (IDA) with high sensitivity mode. The data were pre-processed by MS-DIAL 4.70 software (Tsugawa et al., 2015). The extracted peak information was compared with the MassBank, Respect, and GNPS databases (14,951 records in total).

### 2.3 Animals

SPF-grade healthy male C57BL/6 mice, weighing 18–22 g, were provided by SPEF (Beijing) Biotechnology Co., Ltd. [License No. SCXK (Beijing) 2022–0003]. Mice were housed in a controlled room with a 12 h light/12 h dark cycle at 24 ± 2°C and humidity of 55–70%. All the experiments were approved by the Animal Ethics Committee of Guizhou University of Traditional Chinese Medicine (no. 20220046).

### 2.4 Induction of mice with UC and treatment

After acclimatization for 7 days, mice were randomly assigned to four groups (*n* = 7 per group): control, UC, high-dose, and low-dose AESc groups. According to the clinical application of dosage and the dose conversion from human to mouse, the high-dose and low-dose AESc were set at 2.58 g/kg and 0.65 g/kg, respectively. From days 1 to 14, the AESc groups were orally administered AESc at two concentrations by intragastric gavage respectively, while the control and DSS groups were given pure water. Fresh AESc solution was prepared every other day during dosing. The control group was given pure water during the experiment, and the other groups were allowed free access to 2% (weight/volume) DSS solution for 7 days, and then pure water was given for another 7 days.

### 2.5 General observation and sampling

The disease activity index (DAI) scores were assessed on day 10, and the DAI was calculated using the following formula: DAI = weight loss score + fecal shape score + bloody stool score (Supplementary Table S1). On Day 14, mice were sacrificed by cervical dislocation, and the lengths of the colon were measured. Simultaneously, the liver was weighed, and the organ index was calculated. The colon and liver tissues were immobilized in 4% paraformaldehyde solution for further analyses, and the intestinal contents were snap-frozen and stored at −80°C.

### 2.6 Hematoxylin and eosin stain examination

The colon and liver tissues that had been fixed with 4% paraformaldehyde solution were used and stained by the H&E stain. Histopathological changes were observed and evaluated (Xu et al., 2022). The tissue damage index (TDI) of the colon was scored by combining the depth of the ulcer, degree of inflammatory cell infiltration, and depth of inflammatory cell infiltration (Supplementary Table S2). The tissue damage index (TDI) of the liver was scored by cellular enlargement, cytoplasmic vacuolation, and narrowing of hepatic sinusoids (Supplementary Table S3).

### 2.7 16S rRNA gene sequence analysis

The DNA in the samples was diluted in sterile water, and the genomic DNA was used as a template for PCR amplification using specific primers. The PCR products were detected by electrophoresis and purified using magnetic beads. The purified samples were subjected to a second PCR amplification, followed by electrophoretic detection and magnetic bead purification. The recovered PCR products were quantified by fluorescence using the Quant-iT PicoGreen dsDNA assay kit (BioTek, FLx800, United States). An Agilent high-sensitivity DNA kit was used for quality control of the library on Agilent Bioanalyzer, and sequencing was performed on an Illumina platform. The optimal sequencing length of the selected target fragments was 200–450 bp, and NovaSeq 6,000 SP Reagent Kit (500 cycles) was used for bipartite sequencing on the NovaSeq sequencer. The raw sequences were de-primed, quality filtered, de-noised, spliced, and de-chimerized using the divisive amplicon denoising algorithm 2 (DADA2) method. After the sequencing data were pre-processed into high-quality sequences, the sequences were categorized into multiple operation taxonomic units (OTUs), according to the sequence similarity by using Vsearch software, and the similarity was set at ≥97%. Abundance information, intra-group α-diversity, and inter-group β-diversity of the samples were obtained. Furthermore, the community structure and species composition of the samples were evaluated through multivariate analysis.

SPSS 26.0 software was used for statistical analysis, and the results of each index were described by mean ± standard deviation (SD). The results between groups were analyzed by one-way ANOVA, and the least significant difference (LSD) test was used for a two-by-two comparison. *p* < 0.05 was considered for statistically significant difference. The α-diversity differences between samples were analyzed by Wilcoxon’s rank-order analysis. ^*^p < 0.05 means the difference is statistically significant.

### 2.8 Measurement of SCFAs and a few organic acids in intestinal contents

In total, 5 mg of each intestinal content sample was weighed into 1.5 mL tubes, and 20 μL of water was added and homogenized for 3 min with zirconia beads (B B24, Next Advance, Inc., NY, United States). Overall, 120 μL of methanol-extracted metabolites containing the internal standard was added and centrifuged at 18,000 g for 20 min at 4°C (Microfuge 20R, Beckman Coulter, Inc., IN, United States), and 30 μL of the supernatant was transferred to a 96-well plate. The next steps were performed at the Biomek 4,000 workstation (Beckman Coulter, Inc., Brea, California, United States). In total, 40 μL of freshly prepared derivatization reagent was added to each well, and the plate was sealed and placed at 30°C for 60 min of derivatization (MSC-100, Allsheng Instruments, Co., Ltd., Hangzhou, China). Then, 350 μL of 50% methanol solution was added to an ice bath to dilute the samples. The plate was placed at −20°C for 20 min, followed by centrifugation at 4°C at 4000 g for 30 min. The plate was then centrifuged at 4°C at 4000 g for 30 min, and 135 μL of the supernatant was aspirated and used for LC–MS analysis.

A UPLC–MS/MS system (ACQUITY UPLC-Xevo TQ-S, Waters Corp., Milford, MA, United States) was used for analysis. Elution was performed in a Waters ACQUITY UPLC BEH C_18_ analytical column (2.1 × 100 mm, 1.7 μM) (Waters, USA) at 40°C at a constant flow rate of 0.4 mL/min. The injection volume was 5 μL for each determination. The solvent A was 0.1% formic acid aqueous solution and solvent B was acetonitrile. The elution program was as follows: 0–1 min, 12% B; 1–2.8 min, 12–16.5% B; 2.8–4.2 min, 16.5–24% B; 4.2–5.6 min, 24–30% B; 5.6–8.5 min, 30–100% B; 8.5–10 min, 100% B; 10–11 min, 100–1% B; and 11–12 min, 1–12% B. The ion source temperature was 150°C, and negative ion mode was used to monitor the target ions.

### 2.9 Correlation analysis of metabolites and intestinal microbiota

The correlation between important metabolites and intestinal microbiota was analyzed by Spearman’s correlation coefficient.

### 2.10 Statistical analysis

For pharmacodynamic indicators and short-chain fatty acids, data were presented as mean ± standard error of the mean (SEM) using SPSS software (version 20.0; SPSS Inc., Chicago, Illinois, United States). Means were assessed using ANOVA, and LSD post-hoc test was used to determine the significance of multiple comparisons. *p* < 0.05 was considered for statistically significant difference.

## 3 Results

### 3.1 Analysis of the composition of AESc by LC–MS

The constituents of AESc were determined by UPLC-MS. The MS spectra are shown in Supplementary Figure S2. The retention time (min), accurate mass, and the name of identified components (total score > 95%) in AESc are presented in Supplementary Table S4. All identified components were validated by the accurate fragments. A total of 114 components in AESc had been identified in this study. The AESc components identified were diverse and mainly included triterpenoids, lignans, tannins, and flavonoids.

### 3.2 AESc showed a protective effect on mice with DSS-induced UC

**Figure 1:**
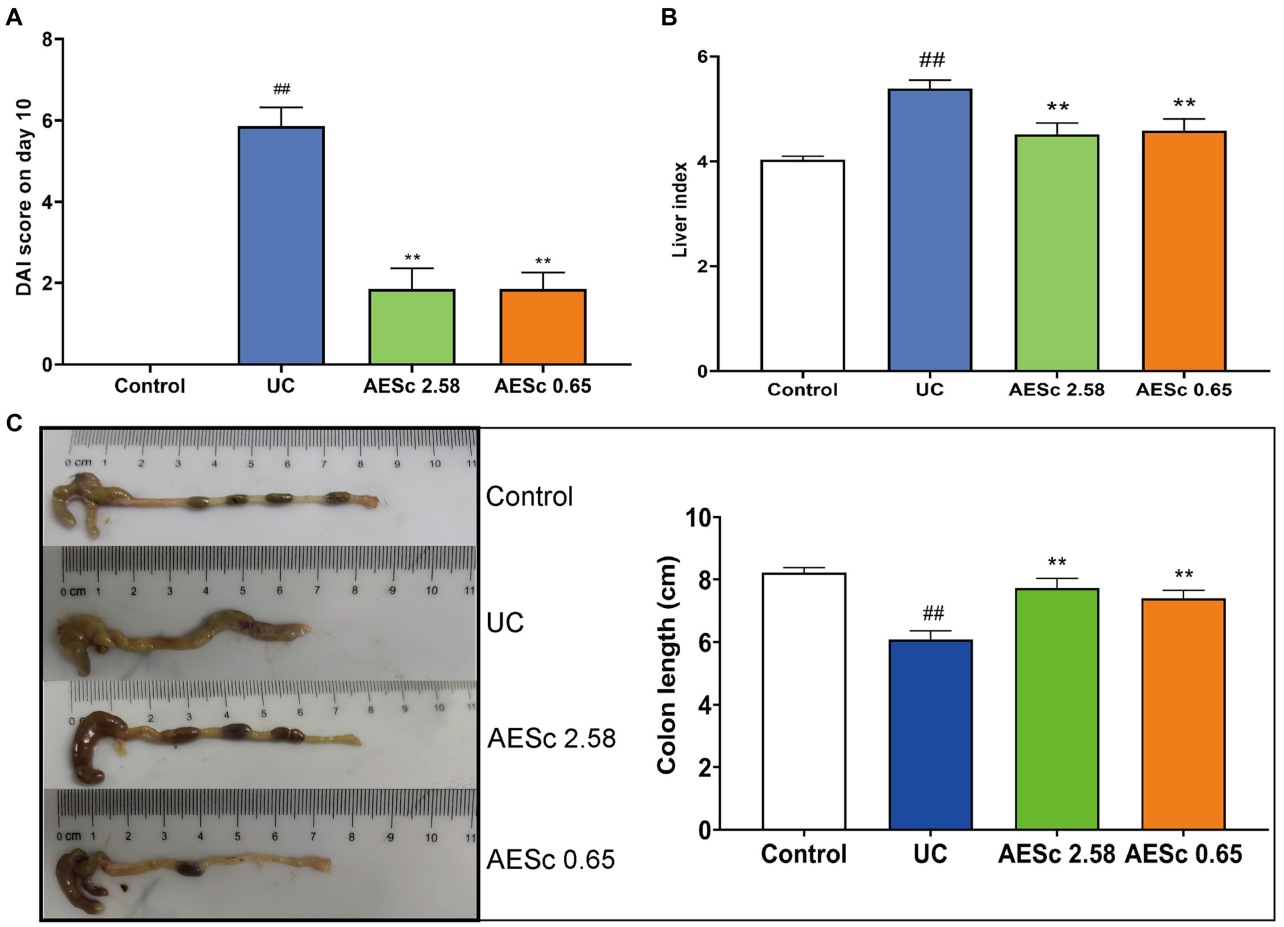
Aqueous extract of *Sargentodoxa cuneata* (AESc) treatment improves disease activity index (DAI) scores, liver index, and colon length in mice with UC. **(A)** DAI scores on day 10 (*n* = 7 per group). **(B)** Effect of AESc on liver index scores in UC mice (*n* = 7 per group). **(C)** The colon length of each group (*n* = 7 per group). The data are represented as mean ± standard error of the mean (SEM). ^#^*p* < 0.05 vs. the control group, ^##^*p* < 0.01 vs. the control group, ^*^p < 0.05 vs. the UC group, ^**^p < 0.01 vs. the UC group.

The body weight of mice in the UC group decreased significantly from days 10 to 13. AESc increased the body weight of mice in the AESc groups. On day 10, AESc significantly decreased the DAI index and liver index of mice with UC (*p* < 0.01). The colon of mice in the UC group was significantly shortened, indicating that an inflammatory response was occurring in the intestines of mice with UC. The AESc groups showed significant improvement in colon length shortening (*p* < 0.01). The results are shown in Figure 1.

### 3.3 AESc attenuated histopathological injuries in mice with DSS-induced UC

**Figure 2:**
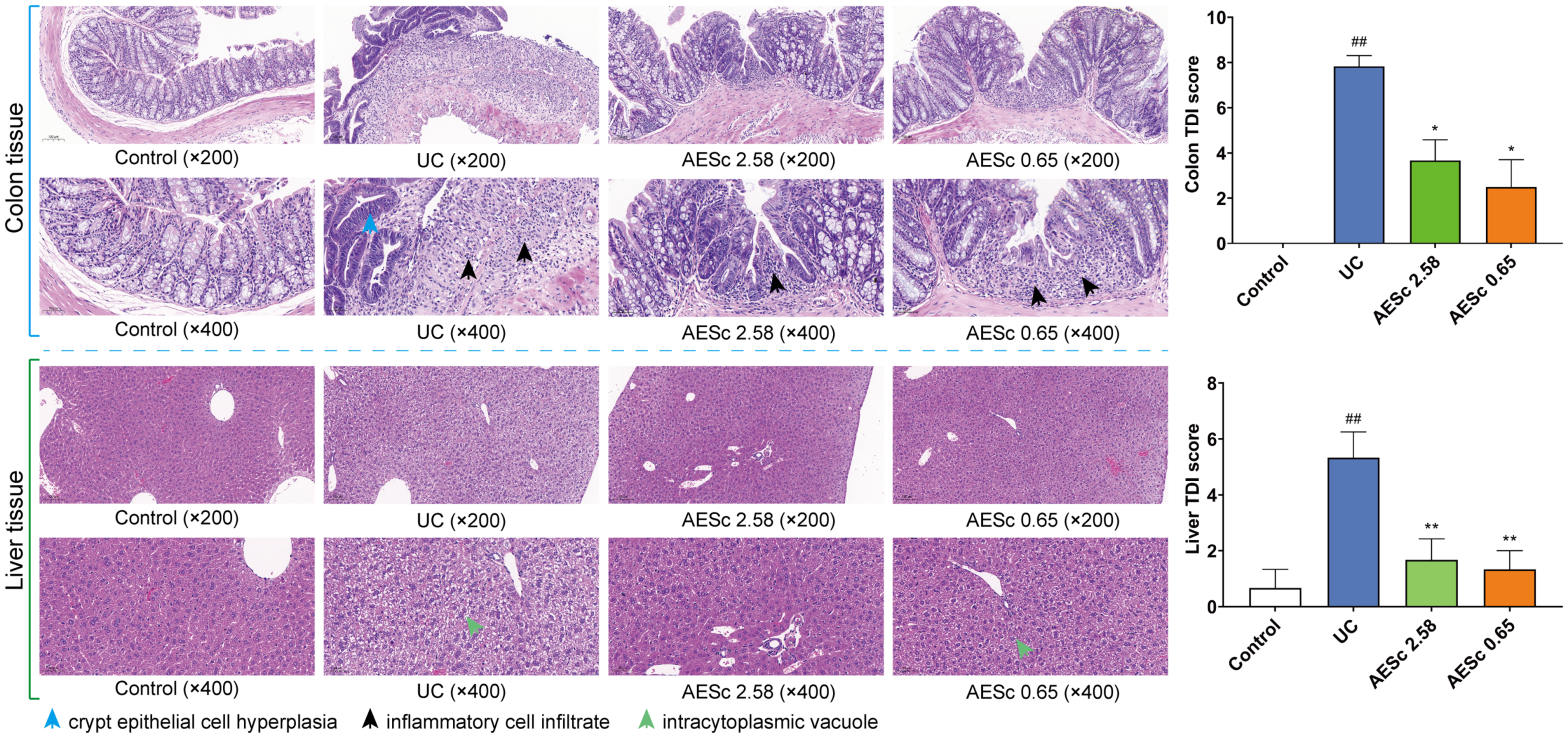
Pathohistological findings of aqueous extract of *Sargentodoxa cuneata* (AESc) on the colon and liver tissue from control, UC, and AESc (2.58 g/kg, 0.65 g/kg) groups and the histological damage index (TDI) (*n* = 6 per group). The data are represented as mean ± standard error of the mean (SEM). ^#^*p* < 0.05 vs. the control group, ^##^*p* < 0.01 vs. the control group, ^*^p < 0.05 vs. the UC group, ^**^p < 0.01 vs. the UC group.

The pathological results of the colon and liver in each group are shown in Figure 2. The colon tissues of mice with UC were damaged in the mucosal layer, submucosal layer, muscular layer, and outer membrane, and infiltrated with a large number of inflammatory cells. In the AESc groups, part of the colon tissues was infiltrated by inflammatory cells, but damage to the mucosal layer, submucosal layer, muscular layer, and outer membrane was ameliorated. The results of the colon TDI score indicated that AESc may treat UC by modulating the inflammatory response and gut barrier (Figure 2). The obvious liver changes in mice with UC were cellular enlargement, cytoplasmic vacuolation, and narrowing of hepatic sinusoids. Furthermore, a part of the liver tissues had a slight inflammatory infiltrate (Figure 2). AESc significantly ameliorated these pathological changes and decreased the liver TDI score, having a protective effect on UC-associated liver injuries.

### 3.4 AESc regulated gut microbiota dysbiosis in mice with DSS-induced UC

**Figure 3:**
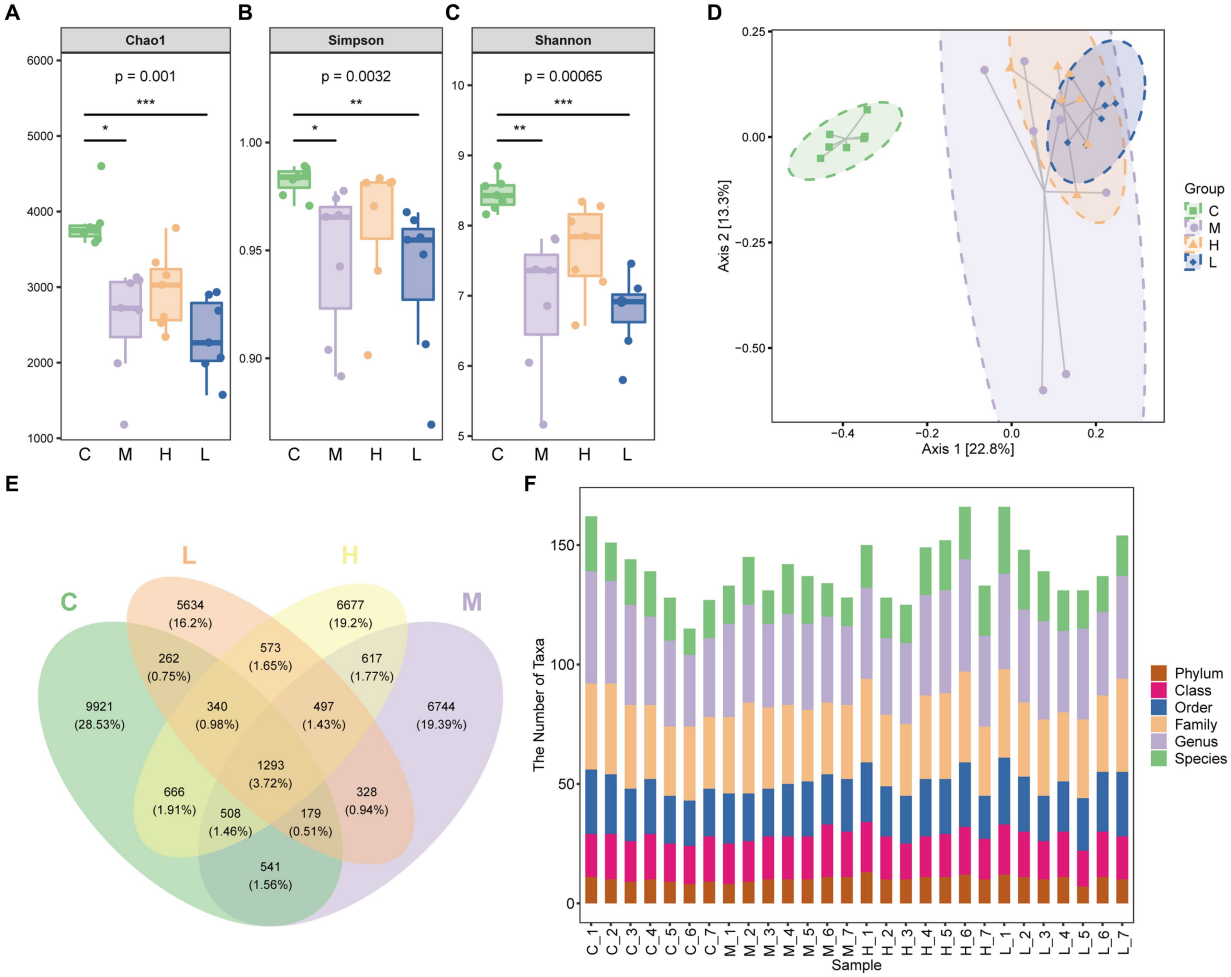
Aqueous extract of *Sargentodoxa cuneata* (AESc) affects the gut flora in mice with DSS-induced UC. **(A)** Chao1 index. **(B)** Simpson index. **(C)** Shannon index. **(D)** PCoA analysis of the intestinal flora of mice in the control group (C), UC group (M), high-dose (H), and low-dose (L) AESc groups. **(E)** The Venn graph and **(F)** the number of taxa of bacterial species at the intergroup phylum, class, order, family, genus, and species levels (*n* = 7 per group). ^*^p < 0.05 vs. the control group, ^**^p < 0.01 vs. the control group, and ^***^p < 0.001 vs. the control group.

The 16S rRNA gene sequence analysis was performed. The Chao1 index, Simpson index, and Shannon index of mice with UC were significantly decreased, indicating a decrease in microbiota diversity (*p* < 0.05 or *p* < 0.01). High-dose AESc enhanced these three indices in mice with DSS-induced UC, while no significant differences were observed between the low-dose AESc group and the UC group (Figures 3A–C). PCoA analysis revealed that there were differences in the composition of the intestinal flora in control, UC, and high-dose, and low-dose AESc groups (Figure 3D). The Venn graph and the number of taxa of bacterial species at the intergroup phylum, class, order, family, genus, and species levels are shown in Figures 3E,F.

**Figure 4:**
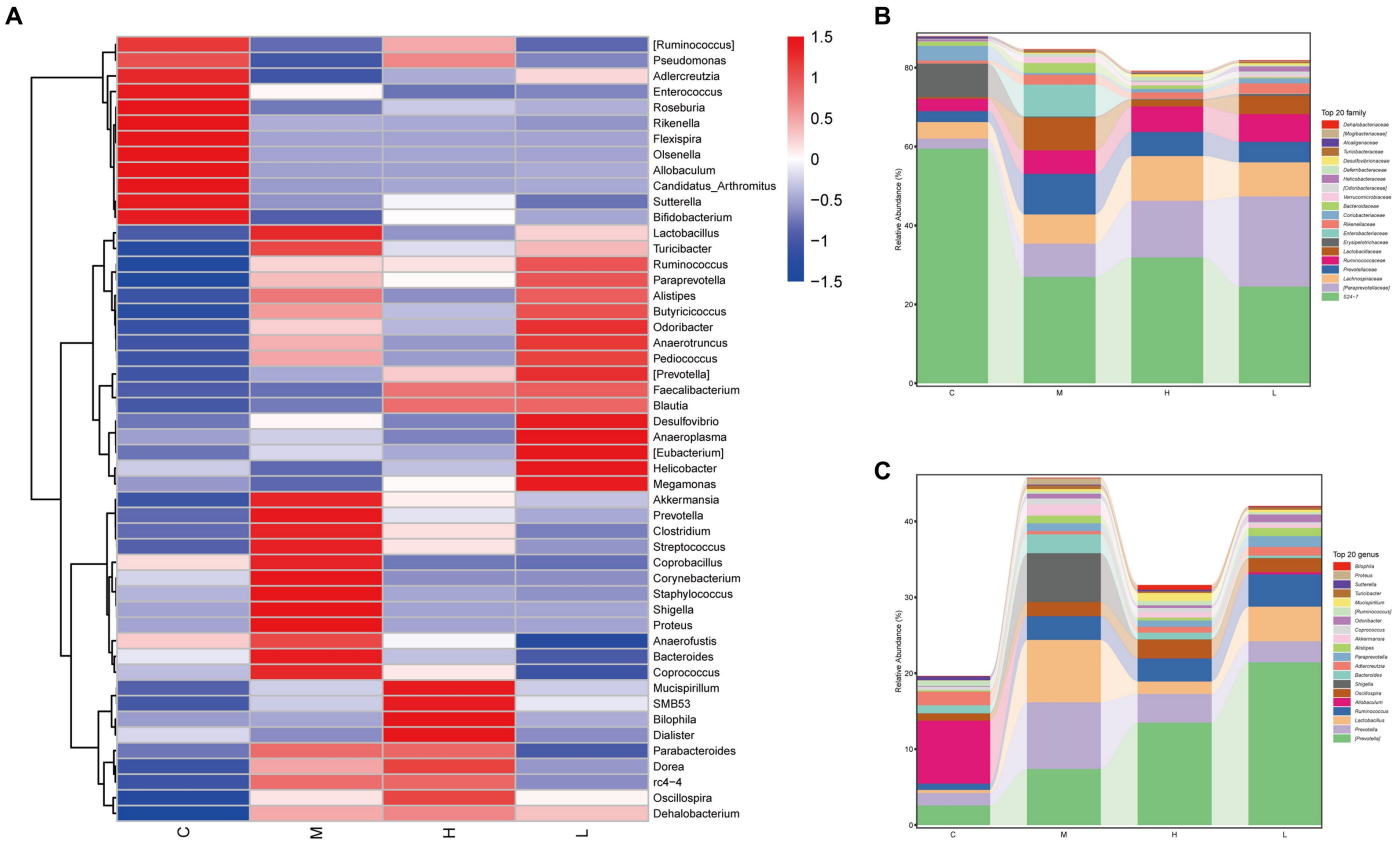
Aqueous extract of *Sargentodoxa cuneata* (AESc) affects the relative abundance of gut flora in mice with DSS-induced UC. **(A)** The heatmap analysis. **(B)** The relative abundance of gut flora categorized by family. **(C)** The relative abundance of gut flora categorized by genus. The control group (C), UC group (M), high-dose AESc group (H), and low-dose AESc group (L) (*n* = 7 per group).

The heatmap also showed some interesting results, and the AESc were able to correct the intestinal dysbiosis of mice with UC, such as *Akkermansia, Prevotella, Corynebacterium, Staphylococcus*, and *Shigella* (Figure 4A). The relative abundance of the top 20 bacterial species at family level and genus level was calculated (Figures 4B,C). AESc increased the relative abundance of Alcaligenaceae, Desulfovibrionaceae, Deferribacteraceae, Helicobacteraceae, Coriobacteriaceae, Ruminococcaceae, Lachnospiraceae, and [Paraprevotellaceae], while decreasing the relative abundance of Turicibacteraceae, Verrucomicrobiaceae, Bacteroidaceae, Enterobacteriaceae, Lactobacillaceae, and Prevotellaceae at the family level in mice with UC (Figure 4B). Categorized by the genus, AESc increased the relative abundance of *Mucispirillum, Adlercreutzia, Oscillospira, Allobaculum, [Ruminococcus]*, and [*Prevotella*], but decreased the relative abundance of *Proteus, Turicibacter, Coprococcus, Akkermansia, Bacteroides, Shigella, Lactobacillus*, and *Prevotella* (Figure 4C).

**Figure 5:**
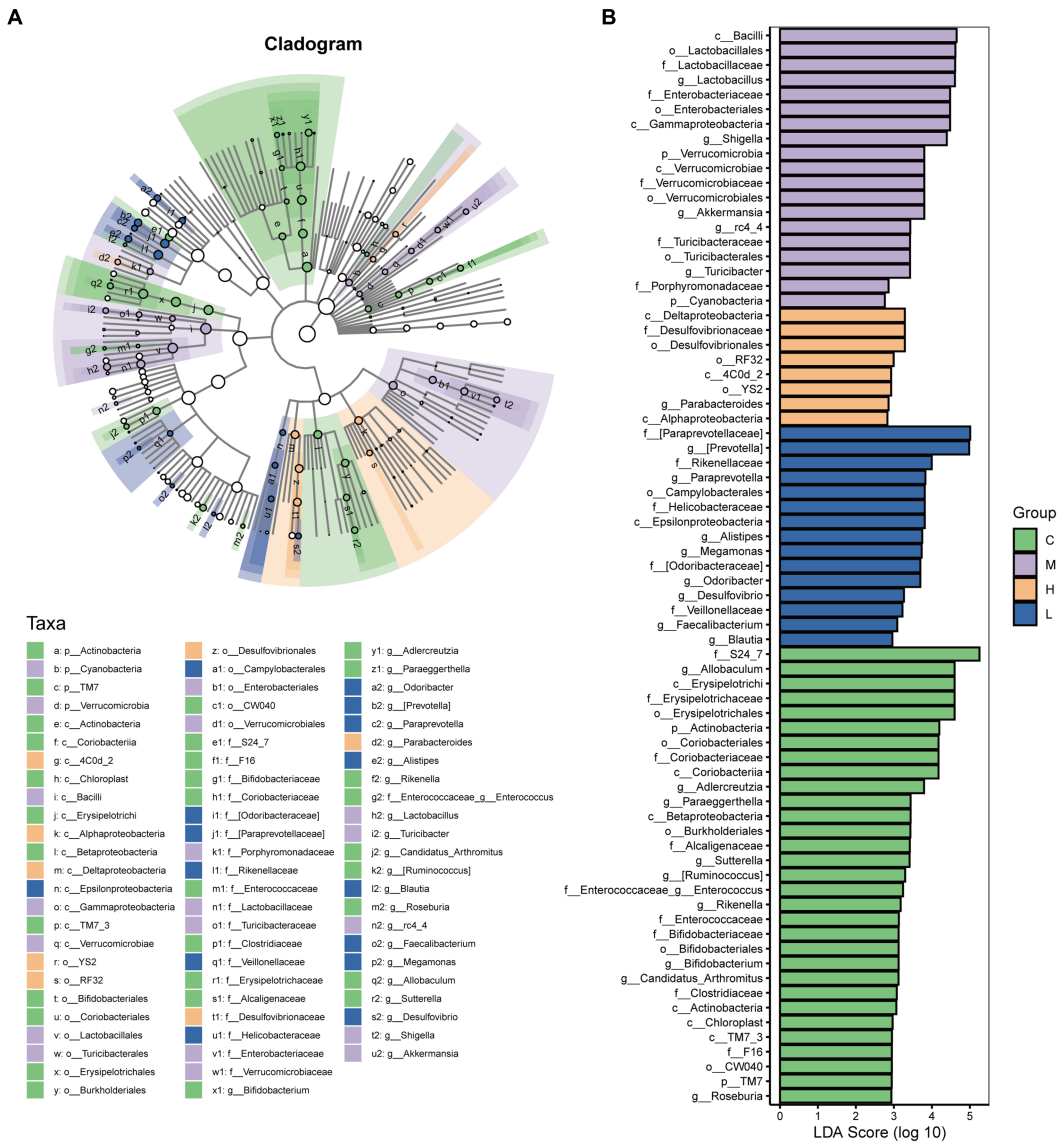
The results of LEfSe analysis. **(A)** The Cladogram results. **(B)** The LDA score (log 10) of each group. The control group (C), UC group (M), high-dose AESc group (H), and low-dose AESc group (L) (*n* = 7 per group).

LEfSe analysis was performed to identify the differential species between groups. A total of 73 taxa were obtained from phylum to genus, including 31 taxa in the control group, 19 taxa in the UC group, 8 taxa in the high-dose AESc group, and 15 taxa in the low-dose AESc group (Figure 5). Differential flora in the high-dose AESc group included Deltaproteobacteria, Desulfovibrionaceae, Desulfovibrionales, RF32, 4C0d_2, YS2, *Parabacteroides*, and Alphaproteobacteria. The differential marker flora in the low-dose AESc group included [Paraprevotellaceae], [*Prevotella*], Rikenellaceae, *Paraprevotella*, Campylobacterales, Helicobacteraceae, Epsilonproteobacteria, *Alistipes, Megamonas*, Odoribacteraceae, *Odoribacter, Desulfovibrio*, Veillonellaceae, *Faecalibacterium*, and *Blautia* (Figure 5B).

### 3.5 AESc affected the levels of SCFAs and a few organic acids in the intestinal contents

**Figure 6:**
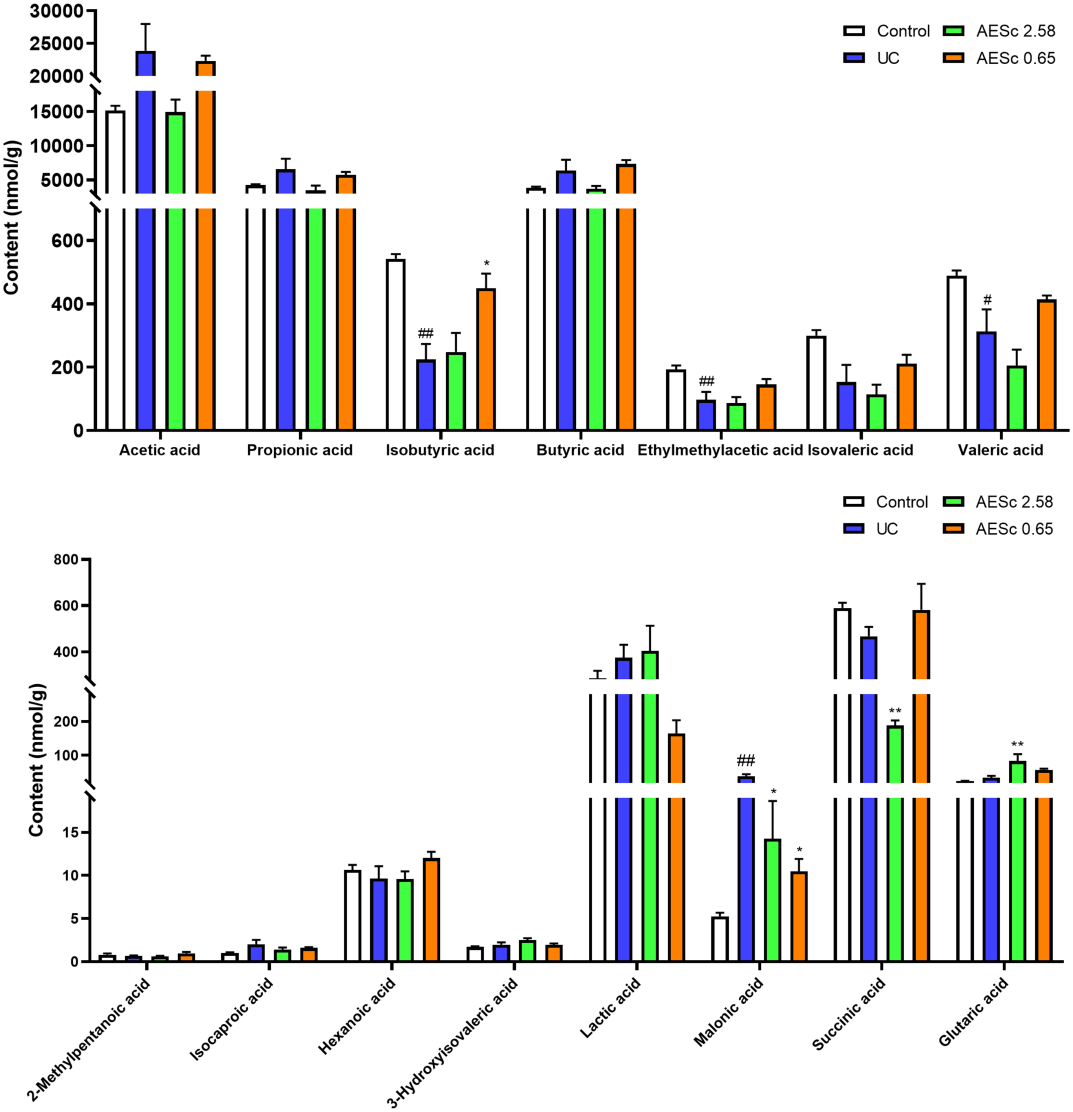
Aqueous extract of *Sargentodoxa cuneata* (AESc) affects the content of SCFAs and organic acids in the intestinal contents in mice with UC (*n* = 6–7 per group). The data are represented as mean ± standard error of the mean (SEM). ^#^*p* < 0.05 vs. the control group, ^##^*p* < 0.01 vs. the control group, ^*^p < 0.05 vs. the UC group, ^**^p < 0.01 vs. the UC group.

The malonic acid in the UC group were significantly increased (*p* < 0.05), while the valeric acid, isobutyric acid, and ethylmethylacetic acid were significantly decreased (*p* < 0.05). High-dose AESc significantly decreased the levels of malonic acid and succinic acid and increased the levels of glutaric acid in the intestinal contents (*p* < 0.05). Low-dose AESc significantly decreased the levels of malonic acid and increased the levels of isobutyric acid in the intestinal contents (*p* < 0.05). The contents of SCFAs in the intestinal contents of mice are shown in Figure 6.

### 3.6 Correlation analysis results of flora and differential SCFAs and organic acids

**Figure 7:**
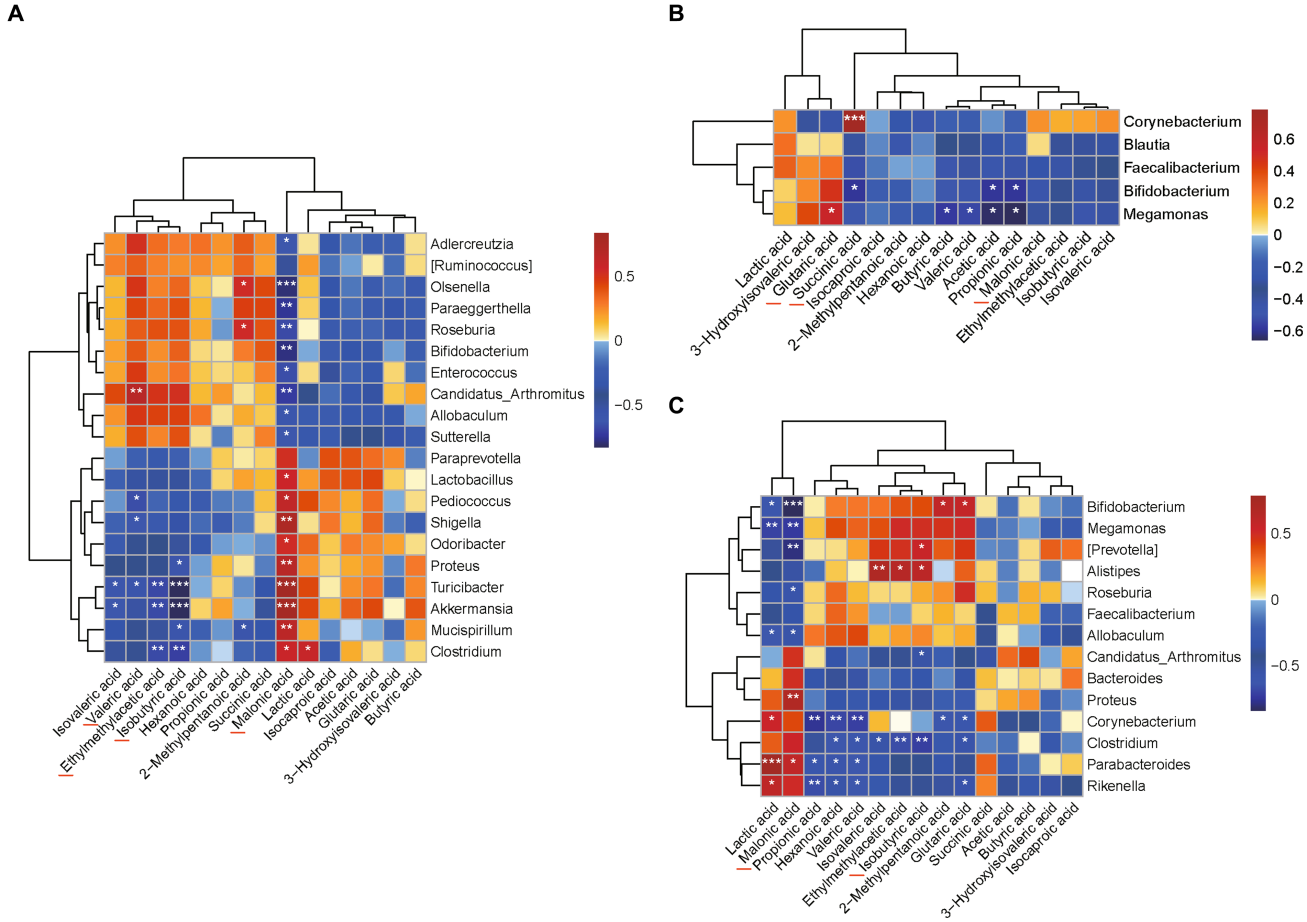
Correlation analysis results between flora and differential SCFAs and some organic acids. **(A)** The results of Spearman’s correlation analysis between the intestinal flora and levels of SCFAs and a few organic acids in the UC group categorized by genus. **(B)** The results of Spearman’s correlation analysis between the intestinal flora and levels of SCFAs and a few organic acids in the high-dose AESc group categorized by genus. **(C)** The results of Spearman’s correlation analysis between the intestinal flora and levels of SCFAs and a few organic acids in the low-dose AESc group categorized by genus. ^*^, ^**^ and ^***^ indicate significant difference at *p* < 0.05, *p* < 0.01, and *p* < 0.001.

Categorized by the genus (Figure 7A), the results of Spearman’s analysis showed that there was a significant correlation between the intestinal flora and SCFAs and some organic acids in the UC group. The levels of glutaric acid and succinic acid in the high-dose AESc group were closely associated with *Megamonas, Bifidobacterium*, and *Corynebacterium* (*p* < 0.05) (Figure 7B). The malonic acid and isobutyric acid in the low-dose AESc group were associated with *Bifidobacterium, Megamonas, [Prevotella], Roseburia, Allobculum, Proteus, Parabacteroides, Alistipes, Candidatus_Arthromitus*, and *Clostridium* (*p* < 0.05) (Figure 7C).

## 4 Discussion

UC is a complex disease resulting in chronic inflammation of the intestinal tract (Le Berre et al., 2023). It is currently believed that immune dysregulation is the key direct pathogenesis of UC, and intestinal flora is an important stimulus for these immune impairment factors (Wastyk et al., 2021). In the present study, we utilized LC-MS to identify 114 compounds with high confidence from AESc, including scopoletin, purpactin A, strophanthidin, convallatoxin, and rhodojaponin V. After the intervention of the AESc, the DAI scores and liver index in mice with UC were improved. Furthermore, AESc also improved the length shortening of the colon and pathological injuries of the colon and liver tissues. AESc had a significant ameliorative effect on mice with UC and UC-associated liver injuries. The results of 16S rRNA gene sequence analysis and metabolomic analysis showed that AESc regulated intestinal flora disorders in mice with UC. Levels of SCFAs and a few organic acids, which are closely associated with intestinal immunoinflammation, were also affected by AESc. In addition, Spearman’s correlation analysis found that the levels of differential SCFAs and a few organic acids were strongly associated with specific gut flora.

The UC seriously jeopardize the patient’s health and cause a series of complications, such as colon carcinogenesis, intestinal hemorrhage, intestinal perforation, arthritis, and cholangitis (Lichtenstein et al., 2022). The UC sequential liver injury is common in clinical practice (Gaspar et al., 2021). The damage to the intestinal mucosal barrier caused by UC allows harmful substances (intestinal flora, lipopolysaccharide, ammonia, etc.) to enter the bloodstream, which leads to liver injuries (Candelli et al., 2021). In this study, AESc improved DAI score and inhibited colon length shortening and colon injuries, suggesting that AESc improved the inflammatory response of UC to play a therapeutic role in the treatment of UC. AESc also reduced the liver index and improved the histopathological changes of the liver in mice with UC. AESc had an ameliorative effect on both UC and UC-associated liver injuries. These results suggest that Chinese medicines can act on multiple pathways to treat UC through multiple components.

Intestinal flora is an indispensable component of the intestinal tract of the body, and the onset and progression of many diseases are associated with intestinal flora (Shuwen and Kefeng, 2022). Studies have found that UC patients have disorders of intestinal flora (Sharma et al., 2022). The 16S rRNA high-throughput analysis was commonly used to reveal the differences in the intestinal flora among the samples (Ma M. et al., 2022). In this study, OTU clustering analysis revealed that there were 13,710 ASV/OTUs in the control group and 11,171 ASV/OTUs in the high-dose AESc group, while 10,707 ASV/OTUs were in the UC group. The diversity of the gut microbiota of UC mice was significantly altered, whereas AESc was able to restore the diversity of the gut flora to some extent. From the values of Chao1, Shannon, and Simmons indices (Figures 3A–C), we concluded that high-dose AESc increased the diversity of gut flora in mice with UC. However, low-dose AESc had less effect on the diversity of the gut microbiota in mice with UC. This might be related to the dose of AESc, the reason for which is uncertain.

Further, PCoA analysis was utilized to analyze the β-diversity of intestinal flora in the intestinal contents of UC mice. PCoA analysis results showed that large differences in the composition of the intestinal flora among the control, UC, and AESc groups were found. The heatmap visualizes the overall gut flora of each group, and the changes in the abundance of intestinal flora reflect the effect of drugs on specific intestinal flora (Cheng et al., 2023). AESc upregulated the abundance of beneficial Alcaligenaceae (Collins et al., 2014), Coriobacteriaceae (Sun et al., 2023), Ruminococcaceae (Keshteli et al., 2022), Lachnospiraceae (Herp et al., 2019), [Paraprevotellaceae], *Mucispirillum* (Herp et al., 2019), *Adlercreutzia* (Chen et al., 2019), *Allobaculum* (van Muijlwijk et al., 2021), [*Ruminococcus*] (Lee et al., 2017), and [*Prevotella*] (Iljazovic et al., 2021) and downregulated the abundance of pathogenic Turicibacteraceae (Tong et al., 2021), Enterobacteriaceae (Hu et al., 2022), *Proteus* (Zhang et al., 2021), *Turicibacter* (Meng et al., 2019), *Coprococcus* (Yang et al., 2023), *Shigella* (Dekker and Frank, 2015), and *Prevotella* (Sharma et al., 2022). However, the modulating effects on Desulfovibrionaceae (Liu et al., 2023), Deferribacteraceae (Berry et al., 2012), Helicobacteraceae (Tong et al., 2021), Verrucomicrobiaceae (Tong et al., 2021), Bacteroidaceae (Peng et al., 2019), Lactobacillaceae (Ma L. et al., 2022), Prevotellaceae (Liu et al., 2020), *Oscillospira* (Chen et al., 2021), *Akkermansia* (Qu et al., 2021), *Bacteroides* (Shao et al., 2021), and *Lactobacillus* (Jia et al., 2022) were inconsistent with the literature reports. We hypothesized that AESc regulates the UC intestinal flora selectively.

Linear discriminant analysis effect size (LEfSe) analysis can be used to discover biomarkers and reveal genomic features, discovering the features in different subgroups that best explain the differences between groups (Zhang et al., 2019). The flora at the phylum, class, order, family, and genus levels were analyzed using LEfSe analysis in the present study. We obtained signature differential flora in each group. The highest number of differential gut microbiota was observed in the control group at the genus level, followed by the UC group. UC caused dysbiosis of the intestinal flora. The differential flora in the control group were predominantly probiotics, including *Allobaculum* (van Muijlwijk et al., 2021), *Adlercreutzia* (Chen et al., 2019), [*Ruminococcus*] (Lee et al., 2017), *Enterococcus* (Růžičková et al., 2020), *Rikenella* (Xu et al., 2022), *Bifidobacterium* (Ishikawa et al., 1955), and *Roseburia* (Luo et al., 2019). The pathogenic bacteria (*Shigella*, *Turicibacter*) were the differential flora in the UC group (Dekker and Frank, 2015; Meng et al., 2019). The differential flora in the high-dose AESc group was probiotic *Parabacteroides* (Ma L. et al., 2022), while that in the low-dose AESc group were *Alistipes* (Wu et al., 2019), *Megamonas* (Walujkar et al., 2018), *Odoribacter* (Osawa et al., 2023), *Faecalibacterium* (Martín et al., 2014), and *Blautia* (Zhuang et al., 2020). In this study, changes in intestinal flora showed a decrease in probiotics and an increase in pathogenic bacteria. AESc was able to regulate disturbed intestinal flora, and special attention can be paid to these differential flora.

The metabolites of intestinal flora include SCFAs and organic acids. In recent years, SCFAs changes have been found to be closely related to the development of UC (Yang et al., 2020). In this study, we quantified 15 intestinal metabolites in the intestinal contents of mice and found that the SCFAs (isobutyric acid, ethylmethylacetic acid, and valerric acid) were significantly decreased in the UC group, while organic acid (malonic acid) was significantly increased. The changes in the levels of SCFAs in mice with UC were consistent with the literature reports (Yang et al., 2020), but, interestingly, we found that AESc significantly reduced the levels of malonic acid. The association of malonic acid with intestinal flora and UC deserves further study. Correlation analyses can initially identify the intestinal flora that influence short-chain fatty acid levels, which lays the foundation for in-depth correlation studies around differential intestinal flora. The correlation of differential SCFAs and a few organic acids with intestinal flora was also explored. *Megamonas* and *Bifidobacterium* were the key intestinal flora related to differential SCFAs and organic acid levels in AESc groups.

Our study had several limitations. First, the composition of AESc was not studied in sufficient depth, and no chemical reference substances were used for validating the studies. Second, despite the observation of histopathological changes in the colon, inflammatory factor changes in the colon of UC mice were not measured. Finally, evidence of the modulating effects of AESc on the specific flora was not provided, and further studies are needed.

## 5 Conclusion

This study demonstrated that AESc alleviated colitis symptoms and protected colon and liver injuries in mice with DSS-induced UC. Furthermore, AESc improved the gut microbiota dysbiosis in mice with UC by increasing the diversity of the intestinal microbiota and the relative abundance of the specific flora. In addition, AESc affected the metabolic levels of SCFAs and a few organic acids in the intestinal contents of mice with UC. *Megamonas* and *Bifidobacterium* were the key intestinal flora related to the differential SCFAs and organic acids. AESc exerts promising protective effects on UC and UC-associated liver injuries. AESc remodels the gut microbiota and regulates the levels of SCFAs and a few organic acids to combat UC.

## Data availability statement

The datasets presented in this study can be found in online repositories. The names of the repository/repositories and accession number(s) can be found in the article/Supplementary material.

## Ethics statement

The animal study was approved by Animal Ethics Committee of Guizhou University of Traditional Chinese Medicine (no. 20220046). The study was conducted in accordance with the local legislation and institutional requirements.

## Author contributions

FX: Conceptualization, Funding acquisition, Investigation, Methodology, Supervision, Validation, Writing – original draft. PY: Investigation, Writing – original draft. HW: Conceptualization, Methodology, Supervision, Writing – review & editing. ML: Investigation, Writing – review & editing. HL: Investigation, Writing – review & editing. QZ: Investigation, Writing – review & editing. DW: Investigation, Writing – review & editing. XW: Conceptualization, Methodology, Supervision, Writing – review & editing.
