# Supplementary material for: Aqueous extract of Sargentodoxa cuneata alleviates ulcerative colitis and its associated liver injuries in mice through the modulation of intestinal flora and related metabolites
Source: Front Microbiol. 2024 Jan 24;15:1295822. doi: 10.3389/fmicb.2024.1295822 (PMC10847537; doi:10.3389/fmicb.2024.1295822)
Supplement: Supplementary file 1 [file Data_Sheet_1.docx]

Supplementary Material

# Supplementary Figures and Tables

## Supplementary Figure


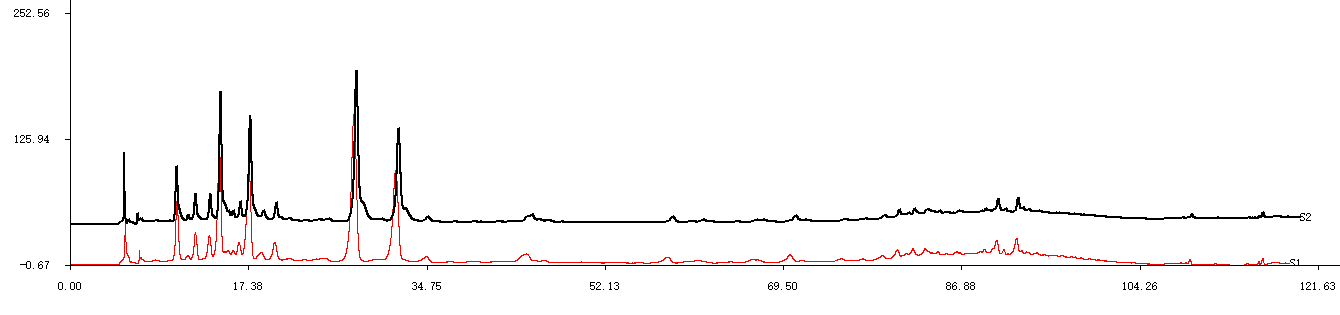


**Supplementary Figure 1.** The stability of the AESc extract was controlled using the high-performance liquid chromatography (HPLC) fingerprint method. S1 similarity=0.998, S2 similarity=0.996. A Waters e2695 HPLC (Waters, USA) equipped with a Diamonsil Plus C_18_ column (4.6 mm × 250 mm, 5 μm) was used. The conditions were as follow: the mobile phase (acetonitrile: methanol = 1:1) was phase A, 0.1% phosphoric acid aqueous solution was phase B. Column temperature was set at 25°C, detection wavelength was 280 nm, flow rate was 0.6 mL/min, and injection volume was 10 μL. Gradient elution: 0-6 min, 7-12% A; 6-40 min, 12-18% A; 40-55 min, 18-20% A; 55-70 min, 20-25% A; 70-85 min, 25-38 % A; 85-100 min, 38-55% A; 100-105min, 55-75% A; 105-110 min, 75-100% A; 110-119 min, 100% A.


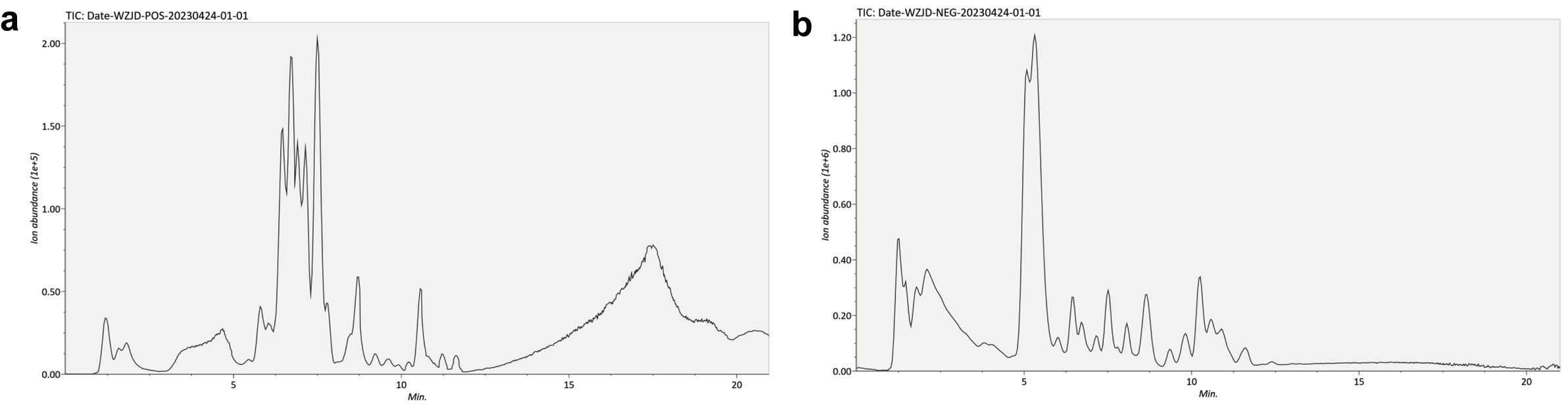


**Supplementary Figure 2.** Total ion chromatogram (TIC) of aqueous extract of *Sargentodoxa cuneata* (AESc). (**a**) TIC in positive ion mode for AESc sample; (**b**) TIC in negative ion mode for AESc sample.

## Supplementary Table

**Supplementary Table 1.** The criteria of disease activity index (DAI) score.

| Score | Weight loss | Fecal shape | Bloody stool |
| --- | --- | --- | --- |
| 0 | 0 | Negative | Negative |
| 1 | 1-5% | Malleable stool | Bloody stool visible to the naked eye (+) |
| 2 | 5-10% | Semi-sloppy stool | ++ |
| 3 | 10-20% | Loose stool | +++ |
| 4 | >20% | Severe loose stool | >+++ |

**Supplementary Table 2.** The criteria of tissue damage index (TDI) of colon.

| Score | Depth of the ulcer | Degree of inflammatory cell infiltration | Depth of inflammatory cell infiltration |
| --- | --- | --- | --- |
| 0 | Negative | Negative | Negative |
| 1 | Epithelium | Mild | Mucosal layer |
| 2 | Mucosal lamina propria | Moderate | Mucosa and submucosa |
| 3 | Mucosal muscularis propria | Severe | Whole colon |

**Supplementary Table 3.** The criteria of tissue damage index (TDI) of liver.

| Score | Cellular enlargement | Cytoplasmic vacuolation | Narrowing of hepatic sinusoids |
| --- | --- | --- | --- |
| 0 | Negative | Negative | Negative |
| 1 | Mild | Mild | Mild |
| 2 | Moderate | Moderate | Moderate |
| 3 | Severe | Severe | Severe |

**Supplementary Table 4.** The composition of aqueous extract of *Sargentodoxa cuneata* (AESc) identified by LC-MS.

| **No.** | **Title** | **RT (min)** | **Precursor m/z** | **Area** | **Adduct** | **Formula** | **Reference m/z** | **Ontology** | **Total score** |
| --- | --- | --- | --- | --- | --- | --- | --- | --- | --- |
| 1 | 2-hydroxy-6-[(8Z,11Z)-pentadeca-8,11,14-trienyl]benzoic acid | 9.498783 | 341.2654 | 223705.2969 | [M-H]^-^ | C_22_H_30_O_3_ | 341.26553 | Salicylic acids | 100 |
| 2 | Rhodojaponin V | 7.10465 | 411.2302 | 99874.46094 | [M+H]^+^ | C_22_H_34_O_7_ | 411.23001 | Grayanoids | 100 |
| 3 | CAPERATIC ACID | 5.947817 | 401.2228 | 98221.04688 | [M-H]^-^ | C_21_H_38_O_7_ | 401.2226 | Tricarboxylic acids and derivatives | 100 |
| 4 | (2R,3R,4S,5S,6R)-2-[(3R)-1,7-bis(3,4-dihydroxyphenyl)heptan-3-yl]oxy-6-(hydroxymethyl)oxane-3,4,5-triol | 7.77365 | 495.2225 | 59652.13672 | [M+H]^+^ | C_25_H_34_O_10_ | 495.22247 | Linear diarylheptanoids | 100 |
| 5 | Citrulline | 6.742833 | 198.0847 | 44832.33203 | [M+H]^+^ | C_6_H_13_N_3_O_3_ | 198.0849 | L-alpha-amino acids | 100 |
| 6 | 2-hydroxy-4-methoxy-3,5-bis(3-methylbut-2-enyl)-6-(2-phenylethyl)benzoic acid | 6.98685 | 431.2193 | 36617.80859 | [M+H]^+^ | C_26_H_32_O_4_ | 431.21927 | Stilbenes | 100 |
| 7 | (2R,3R,4S,6R)-2-((5-hydroxy-2,2-dimethyl-3,4-dihydro-2H-benzo[h]chromen-6-yl)oxy)-6-(hydroxymethyl)tetrahydro-2H-pyran-3,4,5-triol | 6.151767 | 407.166 | 35614.86328 | [M+H]^+^ | C_21_H_26_O_8_ | 407.16599 | Phenolic glycosides | 100 |
| 8 | [(2S,3R,4S,5S,6R)-3,4,5-trihydroxy-6-(hydroxymethyl)oxan-2-yl] (1R,2R,4aS,6aS,6bR,10R,11R,12aR)-1,10,11-trihydroxy-1,2,6a,6b,9,9,12a-heptamethyl-2,3,4,5,6,6a,7,8,8a,10,11,12,13,14b-tetradecahydropicene-4a-carboxylate | 6.3878 | 649.3957 | 33700.50781 | [M-H]^-^ | C_36_H_58_O_10_ | 649.39569 | Triterpene saponins | 100 |
| 9 | 2,3-bis[(4-hydroxy-3-methoxyphenyl)methyl]butane-1,4-diol | 7.305583 | 361.2213 | 27269.88086 | [M-H]^-^ | C_20_H_26_O_6_ | 361.22162 | Dibenzylbutanediol lignans | 100 |
| 10 | (2S,3R,4S,5R)-2-[4-[7-hydroxy-3-(hydroxymethyl)-5-(3-hydroxypropyl)-2,3-dihydro-1-benzofuran-2-yl]-2-methoxyphenoxy]oxane-3,4,5-triol | 7.18315 | 501.1731 | 25077.22266 | [M+H]^+^ | C_24_H_30_O_10_ | 501.1731 | 2-arylbenzofuran flavonoids | 100 |
| 11 | 7-(2-hydroxypropan-2-yl)-1,4a-dimethyl-2,3,4,5,6,7,8,8a-octahydronaphthalen-1-ol | 10.0576 | 239.1381 | 22472.82031 | [M-H]^-^ | C_15_H_28_O_2_ | 239.13782 | Eudesmane, isoeudesmane or cycloeudesmane sesquiterpenoids | 100 |
| 12 | 3-[(2,6-dihydroxy-3,4-dimethylphenyl)methyl]-2,4-dihydroxy-6-methylbenzaldehyde | 11.32618 | 301.0937 | 99156.125 | [M-H]^-^ | C_17_H_18_O_5_ | 301.09421 | Diphenylmethanes | 99.9 |
| 13 | 5-[6-(3-hydroxy-4-methoxyphenyl)-1,3,3a,4,6,6a-hexahydrofuro[3,4-c]furan-3-yl]-2-methoxyphenol | 10.37508 | 357.2598 | 84109.8125 | [M-H]^-^ | C_20_H_22_O_6_ | 357.2594 | Furanoid lignans | 99.9 |
| 14 | (1R,3aR,3bS,4aR,6S,7S,7aR,8R,8aR,9aR)-1,4a,8-tris(acetyloxy)-2,2,3b,6,8a-pentamethyl-4,9-dioxo-tetradecahydro-1H-cyclopenta[a]s-indacen-7-yl butanoate | 8.065367 | 561.27 | 64696.69141 | [M-H]^-^ | C_30_H_42_O_10_ | 561.27051 | Diterpenoids | 99.9 |
| 15 | 2,4-Dihydroxybenzophenone | 5.11465 | 215.0698 | 56227.3125 | [M+H]^+^ | C_13_H_10_O_3_ | 215.07027 | Benzophenones | 99.9 |
| 16 | (2S,3S,4S,8R,9S,13R,14R,15S,16R)-3,4,8,14,15-pentahydroxy-2,13,16-trimethyl-6-methylidene-10-oxatetracyclo[7.6.1.0,.0,]hexadecan-11-one | 5.591 | 367.1758 | 42866.38281 | [M-H]^-^ | C_19_H_28_O_7_ | 367.17621 | Eudesmanolides, secoeudesmanolides, and derivatives | 99.9 |
| 17 | Serine | 8.725734 | 106.0505 | 40695.36719 | [M+H]^+^ | C_3_H_7_NO_3_ | 106.05 | Serine and derivatives | 99.9 |
| 18 | 4-hydroxyphenylacetic acid | 8.607166 | 175.1618 | 34031.41406 | [M+H]^+^ | C_8_H_8_O_3_ | 175.16142 | 1-hydroxy-2-unsubstituted benzenoids | 99.9 |
| 19 | ARTENIMOL | 10.21742 | 283.2094 | 32879.81641 | [M-H]^-^ | C_15_H_24_O_5_ | 283.20984 | Artemisinins | 99.9 |
| 20 | (2R,3R,4R,6aR,6bS,8aS,11R,12R,12aS,14bR)-2,3,12-trihydroxy-4,6a,6b,11,12,14b-hexamethyl-8a-[(2S,3R,4S,5S,6R)-3,4,5-trihydroxy-6-(hydroxymethyl)oxan-2-yl]oxycarbonyl-1,2,3,4a,5,6,7,8,9,10,11,12a,14,14a-tetradecahydropicene-4-carboxylic acid | 6.947783 | 703.367 | 22703.33008 | [M+H]^+^ | C_36_H_56_O_12_ | 703.36639 | Triterpene saponins | 99.9 |
| 21 | formononetin | 6.4678 | 267.1001 | 20173.68555 | [M-H]^-^ | C_16_H_12_O_4_ | 267.0997 | 4'-O-methylisoflavones | 99.9 |
| 22 | [(2R,3S,4S,5R,6S)-6-[3,5-dihydroxy-4-[3-(4-hydroxyphenyl)propanoyl]phenoxy]-3,4,5-trihydroxyoxan-2-yl]methyl 3,4,5-trihydroxybenzoate | 5.474683 | 627.2145 | 18627.14648 | [M+H]^+^ | C_28_H_28_O_14_ | 627.2149 | Flavonoid O-glycosides | 99.9 |
| 23 | [3,4,5-trihydroxy-6-[[(E)-3-(4-hydroxyphenyl)prop-2-enoyl]oxymethyl]oxan-2-yl] 3,4,5-trihydroxybenzoate | 7.146417 | 477.2329 | 1474550 | [M-H]^-^ | C_22_H_22_O_12_ | 477.23224 | Tannins | 99.8 |
| 24 | purpactin A | 1.81805 | 413.1613 | 1002044.063 | [M-H]^-^ | C_23_H_26_O_7_ | 413.16058 | Diarylethers | 99.8 |
| 25 | Isoleucylisoleucine | 8.025367 | 243.1709 | 542965.1875 | [M-H]^-^ | C_12_H_24_N_2_O_3_ | 243.1716 | Peptides | 99.8 |
| 26 | Guanylurea | 6.908633 | 103.0621 | 188741.7656 | [M+H]^+^ | C_2_H_6_N_4_O | 103.06144 | Organic carbonic acids and derivatives | 99.8 |
| 27 | andrastin A | 7.824883 | 485.2917 | 67752.67969 | [M-H]^-^ | C_28_H_38_O_7_ | 485.29239 | Steroid esters | 99.8 |
| 28 | 2-Acetylpyrazine | 7.616183 | 123.0559 | 64516.85938 | [M+H]^+^ | C_6_H_6_N_2_O | 123.0553 | Aryl alkyl ketones | 99.8 |
| 29 | Strictosamide | 5.47285 | 543.1967 | 48838.71094 | [M-H]^-^ | C_26_H_30_N_2_O_8_ | 543.19733 | Carbolines | 99.8 |
| 30 | 4-[3-[(2E)-3,7-dimethylocta-2,6-dienyl]-2,6-dihydroxybenzoyl]-3-formyl-5-hydroxybenzoic acid | 7.705233 | 437.2663 | 38263.49219 | [M-H]^-^ | C_25_H_26_O_7_ | 437.26691 | Benzophenones | 99.8 |
| 31 | [1,3-dihydroxy-1-(7-methoxy-2-oxochromen-6-yl)-3-methylbutan-2-yl] 3-methylbut-2-enoate | 8.741667 | 375.1946 | 36391.875 | [M-H]^-^ | C_20_H_24_O_7_ | 375.194 | Coumarins and derivatives | 99.8 |
| 32 | 1-[4-hydroxy-3-(3-methylbut-2-enyl)phenyl]ethanone | 10.73105 | 203.1084 | 31453.97266 | [M-H]^-^ | C_13_H_16_O_2_ | 203.10774 | Alkyl-phenylketones | 99.8 |
| 33 | 1,3-Dideacetyl-7-Deacetoxy-7-Oxokhivorin | 7.784883 | 457.2592 | 26632.72266 | [M-H]^-^ | C_26_H_34_O_7_ | 457.25858 | Naphthopyrans | 99.8 |
| 34 | 7-[2,6-dimethyl-8-(2-methylbutanoyloxy)-1,2,6,7,8,8a-hexahydronaphthalen-1-yl]-3,5-dihydroxyheptanoic acid | 11.00787 | 421.2602 | 23825.66406 | [M-H]^-^ | C_24_H_38_O_6_ | 421.25955 | Medium-chain hydroxy acids and derivatives | 99.8 |
| 35 | (8aR,12S,12aR)-12-hydroxy-4-methyl-4,5,6,7,8,8a,12,12a-octahydro-1H-3-benzoxecine-2,9-dione | 1.34025 | 251.1283 | 23162.80664 | [M-H]^-^ | C_14_H_20_O_4_ | 251.12888 | Oxocins | 99.8 |
| 36 | Lotaustralin | 6.4685 | 300.0851 | 19314.42773 | [M+H]^+^ | C_11_H_19_NO_6_ | 300.08438 | Cyanogenic glycosides | 99.8 |
| 37 | (7R,8S)-dihydrodehydrodiconiferyl alcohol 9-O-beta-D-glucopyranoside | 7.340683 | 523.2187 | 13988.57813 | [M+H]^+^ | C_26_H_34_O_11_ | 523.21802 | 2-arylbenzofuran flavonoids | 99.8 |
| 38 | [(4E)-7-acetyloxy-6-hydroxy-2-methyl-10-oxo-2,3,6,7,8,9-hexahydrooxecin-3-yl] (E)-but-2-enoate | 11.20653 | 325.2634 | 405127.0625 | [M-H]^-^ | C_16_H_22_O_7_ | 325.26266 | Tricarboxylic acids and derivatives | 99.7 |
| 39 | 3-[[6-[[1,4a-dimethyl-6-methylidene-5-[2-(5-oxo-2H-furan-4-yl)ethyl]-3,4,5,7,8,8a-hexahydro-2H-naphthalen-1-yl]methoxy]-3,4,5-trihydroxyoxan-2-yl]methoxy]-3-oxopropanoic acid | 6.112283 | 605.2349 | 226191.5313 | [M+H]^+^ | C_29_H_42_O_11_ | 605.23584 | Diterpene glycosides | 99.7 |
| 40 | 10-deacetylbaccatin III | 6.825483 | 545.2408 | 165099.5781 | [M+H]^+^ | C_29_H_36_O_10_ | 545.23999 | Taxanes and derivatives | 99.7 |
| 41 | 4-({5-[5-hydroxy-3-({[(2Z)-2-methylbut-2-enoyl]oxy}methyl)pentyl]-8a-(hydroxymethyl)-5,6-dimethyl-3,4,4a,5,6,7,8,8a-octahydronaphthalen-1-yl}methoxy)-4-oxobutanoic acid | 6.825767 | 521.3111 | 153493.0781 | [M-H]^-^ | C_29_H_46_O_8_ | 521.31195 | Colensane and clerodane diterpenoids | 99.7 |
| 42 | 1-Pentanone, 3-hydroxy-1,5-diphenyl- | 6.027983 | 253.1226 | 57870.36328 | [M-H]^-^ | C_17_H_18_O_2_ | 253.1234 | Alkyl-phenylketones | 99.7 |
| 43 | [(3S,4R,5S)-5-[(2S,3R,4S,5S,6R)-4,5-dihydroxy-6-(hydroxymethyl)-2-(4-hydroxyphenoxy)oxan-3-yl]oxy-3,4-dihydroxyoxolan-3-yl]methyl 4-hydroxybenzoate | 5.591 | 523.2641 | 55353.75 | [M-H]^-^ | C_24_H_28_O_13_ | 523.26324 | Phenolic glycosides | 99.7 |
| 44 | 1-[2-[3-[(2R,3R,4S,5S,6R)-6-[[(2R,3R,4R)-3,4-dihydroxy-4-(hydroxymethyl)oxolan-2-yl]oxymethyl]-3,4,5-trihydroxyoxan-2-yl]oxyprop-1-en-2-yl]-2,3-dihydro-1-benzofuran-5-yl]ethanone | 6.947783 | 513.1959 | 42877.39453 | [M+H]^+^ | C_24_H_32_O_12_ | 513.19666 | O-glycosyl compounds | 99.7 |
| 45 | (3S,4S,6aR,6bS,8R,8aR,12aS,14bR)-8-hydroxy-4,6a,6b,11,11,14b-hexamethyl-3-[(2S,3R,4S,5R)-3,4,5-trihydroxyoxan-2-yl]oxy-1,2,3,4a,5,6,7,8,9,10,12,12a,14,14a-tetradecahydropicene-4,8a-dicarboxylic acid | 6.3878 | 633.3685 | 24969.91602 | [M-H]^-^ | C_35_H_54_O_10_ | 633.36749 | Triterpenoids | 99.7 |
| 46 | Digitoxin | 6.547117 | 763.4262 | 23565.49023 | [M-H]^-^ | C_41_H_64_O_13_ | 763.42737 | Cardenolide glycosides and derivatives | 99.7 |
| 47 | (4R,6aS,8aS)-5',6a,8a,9-tetramethyldocosahydrospiro[naphtho[2',1':4,5]indeno[2,1-b]furan-10,2'-pyran]-4-yl butyrate | 10.40863 | 487.3792 | 18617.26172 | [M+H]^+^ | C_31_H_50_O_4_ | 487.38 | Triterpenoids | 99.7 |
| 48 | Scopoletin | 5.0797 | 191.0926 | 1816139.125 | [M-H]^-^ | C_10_H_8_O_4_ | 191.09163 | 7-hydroxycoumarins | 99.6 |
| 49 | (2R,3S,4S,5R,6R)-2-(hydroxymethyl)-6-propan-2-yloxyoxane-3,4,5-triol | 1.5389 | 221.0747 | 1626941.375 | [M-H]^-^ | C_9_H_18_O_6_ | 221.07559 | O-glycosyl compounds | 99.6 |
| 50 | Nicotinamide | 6.908633 | 123.0544 | 197913.3594 | [M+H]^+^ | C_6_H_6_N_2_O | 123.05529 | Nicotinamides | 99.6 |
| 51 | 3-hydroxy-4-(2-hydroxy-6-methylheptan-2-yl)benzoic acid | 1.5389 | 265.1436 | 25657.09961 | [M-H]^-^ | C_15_H_22_O_4_ | 265.14453 | Sesquiterpenoids | 99.6 |
| 52 | 3-Deoxycaryoptinol | 6.428767 | 452.2652 | 19859.83984 | [M+H]^+^ | C_24_H_34_O_7_ | 452.26425 | Furofurans | 99.6 |
| 53 | 3-methoxy-2-(3-methylbut-2-enyl)-5-pentylphenol | 7.625233 | 261.185 | 140086.2656 | [M-H]^-^ | C_17_H_26_O_2_ | 261.186 | Methoxyphenols | 99.5 |
| 54 | (6E,10Z,14E)-6,14-dimethyl-3-methylidene-2-oxo-3a,4,5,8,9,12,13,15a-octahydrocyclotetradeca[b]furan-10-carboxylic acid | 5.591 | 329.1909 | 38145.39063 | [M-H]^-^ | C_20_H_26_O_4_ | 329.19189 | Cembranolides | 99.5 |
| 55 | (2S,3S,4S,5R,6R)-6-[[(3S,6aR,6bS,8aS,14bR)-4,4,6a,6b,11,11,14b-heptamethyl-8a-[(2S,3R,4S,5S,6R)-3,4,5-trihydroxy-6-(hydroxymethyl)oxan-2-yl]oxycarbonyl-1,2,3,4a,5,6,7,8,9,10,12,12a,14,14a-tetradecahydropicen-3-yl]oxy]-3,5-dihydroxy-4-[(2S,3R,4S,5R,6R)-3,4,5-trihydroxy-6-(hydroxymethyl)oxan-2-yl]oxyoxane-2-carboxylic acid | 9.739117 | 955.6807 | 18293.80664 | [M-H]^-^ | C_48_H_76_O_19_ | 955.67883 | Triterpene saponins | 99.5 |
| 56 | 2-[4-hydroxy-2-[(2S,3R,4S,5S,6R)-3,4,5-trihydroxy-6-(hydroxymethyl)oxan-2-yl]oxyphenyl]acetonitrile | 13.66697 | 359.2505 | 16402.13867 | [M-H]^-^ | C_21_H_28_O_5_ | 359.25146 | Naphthopyrans | 99.5 |
| 57 | 9-hydroxy-9-[[(E)-2-(hydroxymethyl)-3-[3-(hydroxymethyl)-6-propan-2-ylcyclohex-2-en-1-yl]prop-2-enoyl]oxymethyl]-1-oxo-6-propan-2-yl-3,5a,6,7,8,9a-hexahydro-2-benzoxepine-4-carboxylic acid | 1.261083 | 533.2744 | 613287.9375 | [M-H]^-^ | C_29_H_42_O_9_ | 533.27557 | Terpene lactones | 99.3 |
| 58 | [(2S,3R,4S,5S,6R)-3,4,5-trihydroxy-6-[[(E)-3-phenylprop-2-enoyl]oxymethyl]oxan-2-yl] 3,4,5-trihydroxybenzoate | 1.261083 | 461.2359 | 53094.83984 | [M-H]^-^ | C_22_H_22_O_11_ | 461.23715 | Tannins | 99.3 |
| 59 | 6-[[5-(hydroxymethyl)-2,5,8a-trimethyl-1,4,4a,6,7,8-hexahydronaphthalen-1-yl]methyl]-3-methylidene-7-oxabicyclo[4.1.0]heptane-2,4,5-triol | 6.98685 | 417.2049 | 30048.45508 | [M+H]^+^ | C_22_H_34_O_5_ | 417.20377 | Oxepanes | 99.3 |
| 60 | (3Z,5E)-4-hydroxy-6-(4-hydroxyphenyl)hexa-3,5-dien-2-one | 8.583834 | 203.0725 | 22379.21484 | [M-H]^-^ | C_12_H_12_O_3_ | 203.07137 | Styrenes | 99.3 |
| 61 | 3-[(3R,5S,10R,13R,14S,17R)-5,14-dihydroxy-10,13-dimethyl-3-[(2R,3R,4S,5S,6R)-3,4,5-trihydroxy-6-(hydroxymethyl)oxan-2-yl]oxy-2,3,4,6,7,8,9,11,12,15,16,17-dodecahydro-1H-cyclopenta[a]phenanthren-17-yl]-2H-furan-5-one | 6.027983 | 551.2847 | 1453351.875 | [M-H]^-^ | C_29_H_44_O_10_ | 551.28613 | Cardenolide glycosides and derivatives | 99.2 |
| 62 | NIACINAMIDE | 8.725734 | 145.0386 | 393054.3438 | [M+H]^+^ | C_6_H_6_N_2_O | 145.03723 | Nicotinamides | 99.1 |
| 63 | Jasmonic acid | 8.1052 | 209.1203 | 21660.20313 | [M-H]^-^ | C_12_H_18_O_3_ | 209.119 | Jasmonic acids | 99.1 |
| 64 | Kuwanon C | 1.5784 | 421.2146 | 51684.53906 | [M-H]^-^ | C_25_H_26_O_6_ | 421.216 | 8-prenylated flavones | 99 |
| 65 | Reserpic acid | 1.37975 | 399.1911 | 22824.55469 | [M-H]^-^ | C_22_H_28_N_2_O_5_ | 399.19254 | Alkaloids | 99 |
| 66 | Chrysanthemyl Alcohol | 6.908633 | 193.1004 | 130414.8281 | [M+H]^+^ | C_10_H_18_O | 193.09892 | Monocyclic monoterpenoids | 98.9 |
| 67 | [3,4,5-trihydroxy-6-[[3,4,5-trihydroxy-6-(hydroxymethyl)oxan-2-yl]oxymethyl]oxan-2-yl] 2,6,6-trimethylcyclohexene-1-carboxylate | 6.746284 | 491.2148 | 56719.26563 | [M-H]^-^ | C_22_H_36_O_12_ | 491.21338 | O-glycosyl compounds | 98.9 |
| 68 | (E)-3-(4-methoxyphenyl)-1-[2,4,6-trimethoxy-3-(3-methylbut-2-enyl)phenyl]prop-2-en-1-one | 7.459167 | 419.2233 | 33715.59375 | [M+H]^+^ | C_24_H_28_O_5_ | 419.22476 | 3-prenylated chalcones | 98.9 |
| 69 | falcarindiol | 7.5454 | 259.1688 | 86659.46875 | [M-H]^-^ | C_17_H_24_O_2_ | 259.17035 | Long-chain fatty alcohols | 98.8 |
| 70 | shogaol | 8.860167 | 275.1668 | 22686.41992 | [M-H]^-^ | C_17_H_24_O_3_ | 275.16525 | Shogaols | 98.8 |
| 71 | (4S,5Z,6S)-4-(2-methoxy-2-oxoethyl)-5-[2-[(E)-3-phenylprop-2-enoyl]oxyethylidene]-6-[(2S,3R,4S,5S,6R)-3,4,5-trihydroxy-6-(hydroxymethyl)oxan-2-yl]oxy-4H-pyran-3-carboxylic acid | 9.498783 | 301.2263 | 19923.96094 | [M-H]^-^ | C_18_H_22_O_4_ | 301.2247 | Dibenzylbutane lignans | 98.8 |
| 72 | 2-methylidene-4-[(2R,3R,4S,5S,6R)-3,4,5-trihydroxy-6-(hydroxymethyl)oxan-2-yl]oxybutanoic acid | 1.261083 | 277.0913 | 59603.57813 | [M-H]^-^ | C_11_H_18_O_8_ | 277.0929 | Saccharolipids | 98.7 |
| 73 | 1-[[(2S,3R,11bR)-3-ethyl-9,10-dimethoxy-2,3,4,6,7,11b-hexahydro-1H-benzo[a]quinolizin-2-yl]methyl]-7-methoxy-3,4-dihydro-2H-isoquinolin-6-one | 7.419683 | 487.255 | 80631.04688 | [M+H]^+^ | C_28_H_36_N_2_O_4_ | 487.25671 | Isoquinolones and derivatives | 98.6 |
| 74 | 4-Hydroxy-6-Methylpyran-2-One | 1.061917 | 126.9836 | 14015.1084 | [M+H]^+^ | C_6_H_6_O_3_ | 126.98528 | Pyranones and derivatives | 98.5 |
| 75 | 10-Hydroxycamptothecin | 1.30075 | 363.1004 | 27647.97461 | [M-H]^-^ | C_20_H_16_N_2_O_5_ | 363.0986 | Camptothecins | 98.4 |
| 76 | Convallatoxin | 1.18175 | 549.2726 | 143587.8281 | [M-H]^-^ | C_29_H_42_O_10_ | 549.27051 | Cardenolide glycosides and derivatives | 98.2 |
| 77 | (2R,3R,4S,5R,6R)-2-[[7-[(2R,3R,4R,5S)-3,4-dihydroxy-5-(hydroxymethyl)oxolan-2-yl]oxy-2-ethenyl-2,4b,8,8-tetramethyl-4,4a,5,6,7,8a,9,10-octahydro-3H-phenanthren-3-yl]oxy]-6-methyloxane-3,4,5-triol | 6.746284 | 581.3354 | 1436422.25 | [M-H]^-^ | C_31_H_50_O_10_ | 581.33313 | Diterpene glycosides | 98.1 |
| 78 | methyl 8-hydroxy-4,5,7,10,14,14-hexamethyl-6,17-dioxo-16-oxapentacyclo[13.2.2.0,.0,.0,]nonadeca-3,7-diene-9-carboxylate | 7.5054 | 441.2829 | 333625.5625 | [M-H]^-^ | C_26_H_34_O_6_ | 441.28088 | Naphthopyrans | 98.1 |
| 79 | 12a-HYDROXY-5-DEOXYDEHYDROMUNDUSERONE | 1.937883 | 341.1514 | 97286.39844 | [M-H]^-^ | C_19_H_18_O_6_ | 341.14932 | Rotenoids | 97.9 |
| 80 | methyl (2R)-2-[(1S,3S,7R,8R,9R,12S,13R)-13-(furan-3-yl)-6,6,8,12-tetramethyl-17-methylidene-5,15-dioxo-2,14-dioxatetracyclo[7.7.1.0,.0,]heptadecan-7-yl]-2-hydroxyacetate | 5.873933 | 509.2124 | 168519.3125 | [M+H]^+^ | C_27_H_34_O_8_ | 509.21457 | Limonoids | 97.8 |
| 81 | 4-[5-[[4-[5-[acetyl(hydroxy)amino]pentylamino]-4-oxobutanoyl]-hydroxyamino]pentylamino]-4-oxobutanoic acid | 5.873933 | 499.2186 | 23950.5957 | [M+H]^+^ | C_20_H_36_N_4_O_8_ | 499.21646 | N-acyl amines | 97.7 |
| 82 | gamma-Glutamylmethionine | 1.261083 | 277.0842 | 65926.625 | [M-H]^-^ | C_10_H_18_N_2_O_5_S | 277.08636 | Dipeptides | 97.6 |
| 83 | Strophanthidin | 6.905766 | 403.2419 | 335050.4063 | [M-H]^-^ | C_23_H_32_O_6_ | 403.24408 | Cardenolides and derivatives | 97.5 |
| 84 | [(1S,3R,3aS,4S,8aR)-1-acetyloxy-3-hydroxy-6,8a-dimethyl-3-propan-2-yl-1,2,3a,4,5,8-hexahydroazulen-4-yl] 4-hydroxybenzoate | 6.746284 | 415.2419 | 79833.07813 | [M-H]^-^ | C_24_H_32_O_6_ | 415.24417 | Sesquiterpenoids | 97.5 |
| 85 | 5-{8(Z),11(Z)-pentadecadienyl}resorcinol | 14.09237 | 315.3153 | 27227.39648 | [M-H]^-^ | C_21_H_32_O_2_ | 315.31754 | Resorcinols | 97.5 |
| 86 | 2-[1-[1-hydroxy-10,13-dimethyl-3-[3,4,5-trihydroxy-6-[[3,4,5-trihydroxy-6-(hydroxymethyl)oxan-2-yl]oxymethyl]oxan-2-yl]oxy-2,3,4,7,8,9,11,12,14,15,16,17-dodecahydro-1H-cyclopenta[a]phenanthren-17-yl]ethyl]-4,5-dimethyl-2,3-dihydropyran-6-one | 7.985367 | 765.4102 | 180629.1719 | [M-H]^-^ | C_40_H_62_O_14_ | 765.40668 | Withanolide glycosides and derivatives | 97.4 |
| 87 | (2R,4'aR,5'S,6'R,6'aS,10'aR,10'bR)-6'-(acetyloxy)-4'a,6'a,10'b-trimethyl-5''-oxo-decahydro-1'H-dispiro[oxirane-2,7'-naphtho[2,1-b]pyran-3',3''-oxolane]-5'-yl 2-methylpropanoate | 4.521567 | 477.2517 | 496779.4375 | [M-H]^-^ | C_26_H_38_O_8_ | 477.24939 | Naphthopyrans | 97.3 |
| 88 | methyl (2E,8E)-9-[3a-hydroxy-6,7-dimethyl-1-(2-methylpropyl)-3-oxo-2,4,7,7a-tetrahydro-1H-isoindol-4-yl]-4,5-dihydroxy-8-methylnona-2,8-dienoate | 6.227467 | 448.2681 | 236575.875 | [M-H]^-^ | C_25_H_39_NO_6_ | 448.27045 | Isoindolones | 97.3 |
| 89 | N-[5-[[4-[4-[acetyl(hydroxy)amino]butylamino]-4-oxobutanoyl]-hydroxyamino]pentyl]-N'-(5-aminopentyl)-N'-hydroxybutanediamide | 7.705233 | 545.3279 | 68725.625 | [M-H]^-^ | C_24_H_46_N_6_O_8_ | 545.33044 | N-acyl amines | 97.3 |
| 90 | 1-methyl-N-(2-(nicotinamido)ethyl)-1H-indole-3-carboxamide | 6.908633 | 323.1523 | 33211.33594 | [M+H]^+^ | C_18_H_18_N_4_O_2_ | 323.14999 | Indolecarboxamides and derivatives | 97.3 |
| 91 | 3-Hydroxysebacic acid | 6.908633 | 219.1204 | 29962.02344 | [M+H]^+^ | C_10_H_18_O_5_ | 219.1227 | Medium-chain hydroxy acids and derivatives | 97.3 |
| 92 | [3,4,5-trihydroxy-6-(hydroxymethyl)oxan-2-yl] 2,4-dihydroxy-6-[(E)-2-phenylethenyl]benzoate | 8.54435 | 417.1984 | 14946.14844 | [M-H]^-^ | C_21_H_22_O_9_ | 417.20068 | Stilbenes | 97.3 |
| 93 | euphodendroidin B | 5.913617 | 737.3185 | 96431.48438 | [M+H]^+^ | C_38_H_50_O_13_ | 737.315 | Jatrophane and cyclojatrophane diterpenoids | 97.2 |
| 94 | Fructose | 5.39435 | 179.0676 | 92633.60156 | [M-H]^-^ | C_6_H_12_O_6_ | 179.07001 | Monosaccharides | 97.2 |
| 95 | [(2R,3S,4S,5R,6R)-6-[(2S,3S,4S,5R)-3,4-dihydroxy-2,5-bis(hydroxymethyl)oxolan-2-yl]oxy-3,4,5-trihydroxyoxan-2-yl]methyl 4-hydroxybenzoate | 7.18315 | 501.1793 | 25317.07031 | [M+H]^+^ | C_19_H_26_O_13_ | 501.1817 | O-glycosyl compounds | 97.2 |
| 96 | (S)-2-((S)-2-(((S)-7-acetamido-1,2,3-trimethoxy-9-oxo-5,6,7,9-tetrahydrobenzo[a]heptalen-10-yl)amino)-4-(methylthio)butanoyl)-6,7-dimethoxy-1,2,3,4-tetrahydroisoquinoline-3-carboxamide | 6.3495 | 735.3065 | 89347.05469 | [M+H]^+^ | C_38_H_46_N_4_O_9_S | 735.31 | Dipeptides | 97.1 |
| 97 | nortrachelogenin | 5.315683 | 373.1917 | 105067.7109 | [M-H]^-^ | C_20_H_22_O_7_ | 373.19415 | Dibenzylbutyrolactone lignans | 97 |
| 98 | DL-Liquiritigenin | 1.30075 | 255.1192 | 22082.06055 | [M-H]^-^ | C_15_H_12_O_4_ | 255.12166 | Flavanones | 97 |
| 99 | (E)-8-(4-hydroxy-6-methoxy-7-methyl-3-oxo-1H-2-benzofuran-5-yl)-2,6-dimethyloct-6-enoic acid | 8.224033 | 361.2376 | 22032.50391 | [M-H]^-^ | C_20_H_26_O_6_ | 361.23511 | Phthalides | 97 |
| 100 | Isoguvacine | 1.816617 | 128.0731 | 44824.67188 | [M+H]^+^ | C_6_H_9_NO_2_ | 128.0706 | Hydropyridines | 96.9 |
| 101 | Decanoic acid | 9.379133 | 171.1364 | 114307.875 | [M-H]^-^ | C_10_H_20_O_2_ | 171.13905 | Medium-chain fatty acids | 96.7 |
| 102 | (3aR,5aR,9bR)-3a-hydroxy-5a,9-dimethyl-3-methylidene-4,5,6,7,8,9b-hexahydrobenzo[g][1]benzofuran-2-one | 6.746284 | 247.167 | 355917.2188 | [M-H]^-^ | C_15_H_20_O_3_ | 247.16972 | Eudesmanolides, secoeudesmanolides, and derivatives | 96.5 |
| 103 | 2,4-bis(3-methylbut-2-enyl)-5-(2-phenylethyl)benzene-1,3-diol | 6.746284 | 349.2146 | 63245.91406 | [M-H]^-^ | C_24_H_30_O_2_ | 349.21729 | Stilbenes | 96.5 |
| 104 | Tetrahydrogambogic Acid | 1.77645 | 671.2468 | 35210.75 | [M+H]^+^ | C_38_H_48_O_8_ | 671.25043 | Pyranoxanthones | 96.5 |
| 105 | arctigenin | 8.224033 | 371.2078 | 25626.18945 | [M-H]^-^ | C_21_H_24_O_6_ | 371.20514 | Dibenzylbutyrolactone lignans | 96.5 |
| 106 | (1S,3R,17S,19S)-3-hydroxy-9,9,16,16-tetramethyl-8-oxa-14,23,25-triazaheptacyclo[17.5.2.0,.0,.0,.0,.0,]hexacosa-4,6,10,12,14-pentaene-24,26-dione | 6.107316 | 446.2164 | 100122.4922 | [M-H]^-^ | C_26_H_29_N_3_O_4_ | 446.21927 | 2,2-dimethyl-1-benzopyrans | 96.1 |
| 107 | (2R,3S,4S,5R,6S)-2-(hydroxymethyl)-6-[4-[(E)-3-hydroxyprop-1-enyl]phenoxy]oxane-3,4,5-triol | 9.3393 | 311.1164 | 90394.15625 | [M-H]^-^ | C_15_H_20_O_7_ | 311.11362 | Phenolic glycosides | 96.1 |
| 108 | Tetrahydrosappanone A Trimethyl Ether | 1.658233 | 329.1518 | 550783.625 | [M-H]^-^ | C_19_H_22_O_5_ | 329.14896 | Homoisoflavans | 96 |
| 109 | 3-(3,4-dihydroxyphenyl)-5,7-dihydroxy-6,8-bis(3-methylbut-2-enyl)chromen-4-one | 6.227467 | 421.2431 | 50942.23047 | [M-H]^-^ | C_25_H_26_O_6_ | 421.24014 | 6-prenylated isoflavanones | 95.8 |
| 110 | (1S,2R,4aS,6aR,6bR,10S,12aR)-10-[(2R,3R,4S,5R,6S)-3,4-dihydroxy-6-methyl-5-[(2S,3R,4S,5S,6R)-3,4,5-trihydroxy-6-(hydroxymethyl)oxan-2-yl]oxyoxan-2-yl]oxy-1,2,6b,9,9,12a-hexamethyl-2,3,4,5,6,6a,7,8,8a,10,11,12,13,14b-tetradecahydro-1H-picene-4a,6a-dicarboxylic acid | 7.625233 | 793.4447 | 60430.62891 | [M-H]^-^ | C_42_H_66_O_14_ | 793.43994 | Triterpene saponins | 95.6 |
| 111 | gamma-mangostin | 7.5054 | 395.2672 | 96909.79688 | [M-H]^-^ | C_23_H_24_O_6_ | 395.2641 | 8-prenylated xanthones | 95.5 |
| 112 | 2-acetyl-6-[(3-butanoyl-2,4,6-trihydroxy-5-methylphenyl)methyl]-3,5-dihydroxy-4,4-dimethylcyclohexa-2,5-dien-1-one | 5.119033 | 417.2219 | 81062.53906 | [M-H]^-^ | C_22_H_26_O_8_ | 417.21881 | Alkyl-phenylketones | 95.4 |
| 113 | [(1S,2R,4aR,8aR)-1-acetyloxy-1,4a-dimethyl-6-oxo-7-propan-2-ylidene-2,3,4,5,8,8a-hexahydronaphthalen-2-yl] 3-acetyloxy-2-hydroxy-2-methylbutanoate | 1.419567 | 451.2306 | 66634.23438 | [M-H]^-^ | C_24_H_36_O_8_ | 451.23373 | Sesquiterpenoids | 95.2 |
| 114 | 1-[2,4-dihydroxy-3-[(2S,3R,4R,5S,6R)-3,4,5-trihydroxy-6-(hydroxymethyl)oxan-2-yl]phenyl]-2-hydroxy-3-(4-hydroxyphenyl)propan-1-one | 1.618233 | 435.2335 | 22829.34961 | [M-H]^-^ | C_21_H_24_O_10_ | 435.23032 | 2'-Hydroxy-dihydrochalcones | 95.2 |
